# Enhancement of the Antioxidant and Antimicrobial Activities of Porphyran through Chemical Modification with Tyrosine Derivatives

**DOI:** 10.3390/molecules26102916

**Published:** 2021-05-14

**Authors:** Pedro Adão, João Reboleira, Marco Teles, Beatriz Santos, Nádia Ribeiro, Carlos M. Teixeira, Mafalda Guedes, João Costa Pessoa, Susana Bernardino

**Affiliations:** 1 MARE, Politécnico de Leiria, Edifício CETEMARES, Av. Porto de Pesca, 2520-630 Peniche, Portugal; joao.reboleira@ipleiria.pt (J.R.); 4190821@my.ipleiria.pt (M.T.); beatrizipdossantos@gmail.com (B.S.); 2 Centro Química Estrutural and Departamento de Engenharia Química, Instituto Superior Técnico, Universidade de Lisboa, Av. Rovisco Pais, 1049-001 Lisboa, Portugal; nadia.ribeiro@tecnico.ulisboa.pt (N.R.); cmbt1984@gmail.com (C.M.T.); joao.pessoa@tecnico.ulisboa.pt (J.C.P.); 3 CDP2T and Department of Mechanical Engineering, Escola Superior de Tecnologia de Setúbal, Instituto Politécnico de Setúbal, 2910-761 Setúbal, Portugal; mafalda.guedes@estsetubal.ips.pt; 4 CeFEMA, Instituto Superior Técnico, Universidade de Lisboa, Av. Rovisco Pais, 1049-001 Lisboa, Portugal; 5 MARE, Escola Superior de Turismo e Tecnologia do Mar, Politécnico de Leiria, Av. Porto de Pesca, 2520-630 Peniche, Portugal; susana.bernardino@ipleiria.pt

**Keywords:** modified porphyran, antioxidant activity, antimicrobial activity

## Abstract

The chemical modification of porphyran hydrocolloid is attempted, with the objective of enhancing its antioxidant and antimicrobial activities. Sulfated galactan porphyran is obtained from commercial samples of the red algae Porphyra dioica using Soxhlet extraction with water at 100 °C and precipitation with isopropyl alcohol. The extracted porphyran is then treated with modified L-tyrosines in aqueous medium in the presence of NaOH, at ca. 70 °C. The modified tyrosines L1 and L2 are prepared through a Mannich reaction with either thymol or 2,4-di-tert-butylphenol, respectively. While the reaction with 2,4-di-tert-butylphenol yields the expected tyrosine derivative, a mixture of products is obtained with thymol. The resulting polysaccharides are structurally characterized and the respective antioxidant and antimicrobial activities are determined. Porphyran treated with the N-(2-hydroxy-3,5-di-tert-butyl-benzyl)-L-tyrosine derivative, POR-L2, presents a noticeable superior radical scavenging and antioxidant activity compared to native porphyran, POR. Furthermore, it exhibited some antimicrobial activity against *S. aureus.* The surface morphology of films prepared by casting with native and modified porphyrans is studied by SEM/EDS. Both POR and POR-L2 present potential applicability in the production of films and washable coatings for food packaging with improved protecting characteristics.

## 1. Introduction

Red algae are a well-established source of economically relevant materials such as the galactan polysaccharides. Of these polysaccharides, the sulfated galactans known as carrageenans are widely used as thickening agents in the food industry. In addition to its thickening properties, carrageenans have several useful characteristics that allow its application beyond the scope of the food industry. For instance, it is known that carrageenan exhibits antioxidant and antimicrobial activity [1,2,3,4,5]. Furthermore, carrageenan finds potential use as a structural matrix in biomedical and catalytic applications [6,7,8,9,10,11,12].

Another sulfated galactan of interest is porphyran, which is found in the red algae of the *Porphyra* genus. This genus of red macroalgae contains several species which are typically used for human consumption in European and Eastern European diets (laver, nori) and, thus, are a readily available and potentially renewable source for this polysaccharide. Porphyrans are structurally related to carrageenans, in the sense that porphyrans are linear galactose polymers consisting of 3-linked β-d-galactose units alternating with 6-O-methyl-α-d-galactose, 4-linked α-l-galactose-6-sulfate, or 3,6-anhydro-α-l-galactose units [13,14,15,16]. As a result, carrageenan and porphyran can exhibit similar antioxidant and antimicrobial activities, which have been linked, at least in part, to the galactose sulfate ester units that make up the galactan chain [17]. For instance, porphyran obtained from nori was reported to exhibit some antioxidant activity against superoxide and hydroperoxyl radicals [18]. Furthermore, biological activity has also been reported for porphyran, with this polysaccharide presenting some antiviral [19] and antifungal [20] activity. As such, porphyran has been the focus of recent research efforts concerning the study of its properties and potential applications, namely to produce films and coatings for food packaging.

Owing to the apparent structure-activity relationship observed in porphyran, the direct chemical modification of this polysaccharide has been recently attempted with the purpose of enhancing antioxidant activity [17,21,22,23,24]. In alternative, other methodologies are available for the enhancement of the antioxidant and/or antimicrobial activities of galactan-type polysaccharides, such as agarose, porphyrin, or carrageenan. The most straightforward method involves the simple incorporation by entrapment of an active compound within the polysaccharide matrix [25]. The immobilization of compounds with antimicrobial and antioxidant activity onto polysaccharides takes special relevance in the development of environmentally friendly protective coatings and films, such as active packaging for highly perishable fresh foods [26,27,28,29,30,31].

Despite the convenience of the entrapment method, there is the possibility that the active compound may leach from the polysaccharide matrix [29,30,31,32,33]. While the controlled release of an active compound can be useful in drug delivery applications, the release of active compounds onto foodstuffs is not a desirable aspect in the development of protective films and coatings. In this case, the covalent immobilization of the active compound onto the polysaccharide matrix can minimize the release of the incorporated active compounds, and subsequently minimize the contamination of the foodstuff being protected.

The present work describes the derivatization of porphyran by taking advantage of the electrophilic character of the sulfate ester moieties, with the objective of enhancing its antioxidant and/or antimicrobial properties. This electrophilic character should enable the covalent grafting of suitable small molecules directly onto the polymer chain through a S_N_2 reaction [34]. In turn, this reactivity should enable the direct covalent immobilization of numerous naturally occurring and biologically relevant compounds with antimicrobial and antioxidant activities. For instance, naturally occurring phenols such as thymol, carvacrol and 2,4-di-tert-butylphenol are interesting naturally occurring compounds known for their antimicrobial and antioxidant activities [35,36,37,38,39,40]. Another class of compounds of interest are amino acids, due to their relevant biological role and structural diversity. Amino acids such as tyrosine and cysteine can be chemically modified using straightforward methodologies [41,42,43,44,45] and provide sufficiently reactive functional side chains that can bind covalently onto the porphyran chain.

The literature regarding the chemical modification of porphyran is still scarce, thus this work is an attempt to widen the scope of application of novel modified porphyrans with antioxidant and/or antimicrobial activity. In turn, this may allow the valorization of this widely consumed polysaccharide by enabling its application as potential protective coatings and films for perishable foodstuffs.

## 2. Results and Discussion

### 2.1. Extraction of Porphyran (POR) from Porphyra dioica

Native porphyran (POR) was extracted from the red algae *Porphyra dioica* using adaptations of methods reported in the literature [13]. In this case, porphyran was extracted from the powdered biomass with hot water, using a Soxhlet extractor. The extraction process was run for a minimum of 7 h to ensure optimal yields. The obtained liquid extract was filtered hot with a filter paper under vacuum, evaporated, and precipitated with isopropyl alcohol to yield native porphyran as a light brown solid. The obtained solid was characterized by FTIR, UV–vis and elemental analysis. The characterization of POR and its modified variants is discussed in the following sections.

### 2.2. Synthesis of Modified Tyrosine

**Figure 1:**
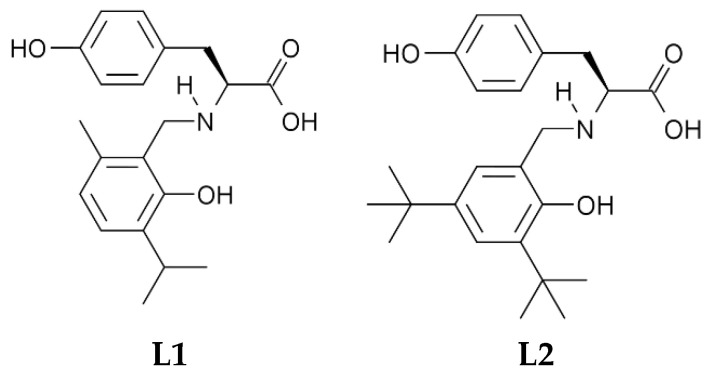
Proposed structural formulas of the L-tyrosine derivatives **L1** and **L2**.

The amino acid L-tyrosine was the initial compound of interest as a derivatizing agent, since the phenolic side chain can be used as an anchoring point to the polysaccharide chain, under the appropriate conditions (Figure 1). The N-substituted derivatives of this amino acid were prepared through a selective multicomponent, single-step Mannich reaction with an appropriate substituted phenol such a thymol or 2,4-di-*tert*-butylphenol (Scheme S1). These phenols were chosen for the reactive sites available for electrophilic substitution, thus minimizing the chances for parallel condensation reactions to occur. Furthermore, both thymol and 2,4-di-*tert*-butylphenol were shown to exhibit antimicrobial activity, in addition to the expected antioxidant potential typical of phenols [35,36,37,38,39,40].

The selectivity of this reaction towards the formation of the desired product relied on the different nucleophilic characters of the disubstituted phenols and the side chain of tyrosine. It can be expected that the higher degree of substitution of thymol and 2,4-di-*tert*-butylphenol with electron-donating alkyl groups would render these compounds more electron-rich than the monoalkylphenol moiety of tyrosine. The electron density on the aromatic ring can be gauged by the pKa of the phenolic hydroxyl group, as the increased aromatic ring substitution with electron-donating alkyl groups is reported to increase the pKa of the phenol [46,47,48,49]. In turn, a higher pKa can indicate an increased reactivity of the phenol towards electrophilic aromatic substitution reactions, [50,51,52] as is the case of the phenolic Mannich reaction [53].

The reported pKa for the phenolic hydroxyl of tyrosine is ca. 9.7 [54], while for thymol and 2,4-di-*tert*-butylphenol is ca. 10.6 and 11.6, respectively [55]. This suggests that these disubstituted phenols should be more reactive towards electrophilic substitution than the tyrosine phenol [56,57]. For instance, it can be expected that thymol and 2,4-di-*tert*-butylphenol would react faster with the imine electrophile than the phenol moiety of tyrosine and limit the self-condensation of tyrosine. To minimize this occurrence, formaldehyde was the last reagent to be added to the reaction mixture. The objective of this reagent addition order was to favor the rapid scavenging of the disodium tyrosinate imine electrophile upon its formation by the disubstituted phenols [53].

The Mannich reaction between disodium L-tyrosinate and thymol to produce **L1** yielded a light-brown solid, which was isolated after the adjustment of pH reaction medium pH to ca. 2 with HCl. This allowed the removal of unreacted tyrosine, its self-condensation products, and the unreacted phenol by thorough washing with water, diethyl ether and/or petroleum ether. However, the reaction product yielded very complex NMR spectra (see Figure S1) and its structure could not be properly elucidated. This was attributed to polycondensation reactions between thymol and tyrosine, given that thymol has *ortho* and *para* positions available for reaction. As a result, no further attempts of syntheses of involving thymol were carried out.

In contrast, the resulting Mannich reaction product between disodium L-tyrosinate and 2,4-di-*tert*-butylphenol was isolated as a water-insoluble off-white solid (**L2**) which could be characterized by NMR. Bulk analysis by obtained EA was consistent with the formulation expected for **L2** with residues of water.

The regioselectivity of this reaction was checked by NMR (Figures S2–S4), namely by heteronuclear multiple bond correlation (HMBC). The HMBC spectrum of **L2** in acidic acetone presented 3-bond correlations which indicated that the reaction proceeded with the desired regioselectivity [53].

### 2.3. Treatment of Porphyran with the Modified Tyrosines

**Figure 2:**
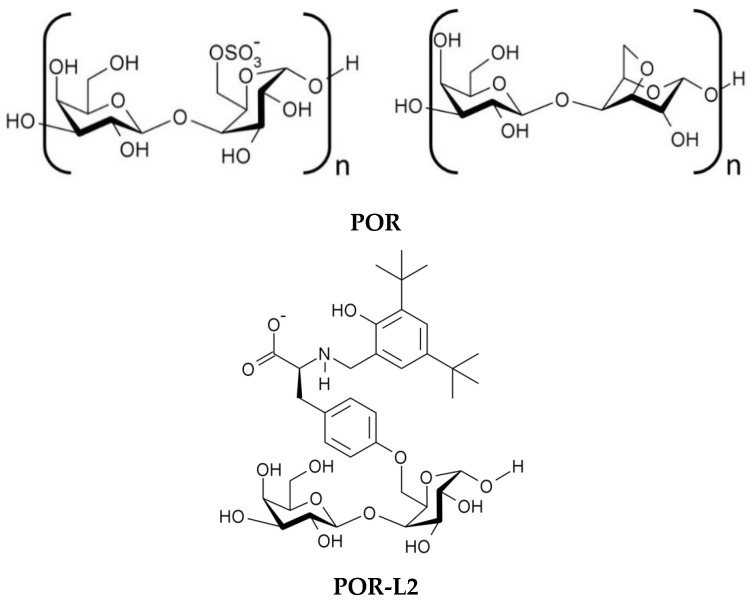
Structural formulas of the main repeating units of **POR** and the proposed structural formula of the modified porphyran **POR-L2**.

Native porphyran (**POR**) was treated with precursor **L2** in the presence of KOH to afford the intended modified variant **POR-L2** (Figure 2), according to the reaction outline presented in Scheme S2. While monoalkylsulfates are less reactive than the dialkyl counterparts, the reactivity of the primary sulfate ester groups in sulfated galactans such as carrageenan and porphyran is sufficient to allow the intramolecular S_N_2 *O*-alkylation reaction under alkaline extraction conditions, driving the formation of the 3,6-anhydrogalactose units in the polymer chain (Scheme S3) [58,59,60].

Thus, it can be anticipated that by reacting strongly nucleophilic species with the primary sulfate ester moieties present in the polymer chain, it would be possible to replace these labile sulfate moieties with the selected nucleophile to some extent. For the preparation of **POR-L2**, the nucleophile used was **L2**^2−^, in which the tyrosine side chain was selectively deprotonated with a suitable alkali metal hydroxide (KOH). The selective deprotonation should be possible due to the different pKa values of the side chain and 2,4-di-*tert*-butylphenol moieties (9.7 versus 11.6, respectively). For the S_N_2 substitution reaction to occur, **L2**^2−^ must approach the galactose-6-sulfate units in the polysaccharide chain. However, the overall steric bulk of **L2**^2−^ could be a hindrance in this regard and slow the nucleophilic substitution reaction. To account for this factor, the reaction was carried out for 24 h to ensure that the substitution reaction occurred to some observable extent. The obtained product was washed thoroughly with isopropyl alcohol to remove unreacted **L2**^2−^ and was characterized by Fourier transform infrared spectroscopy (FTIR), UV–vis, NMR, SEM, DSC, and elemental analysis.

#### 2.3.1. FTIR Analysis

**Figure 3:**
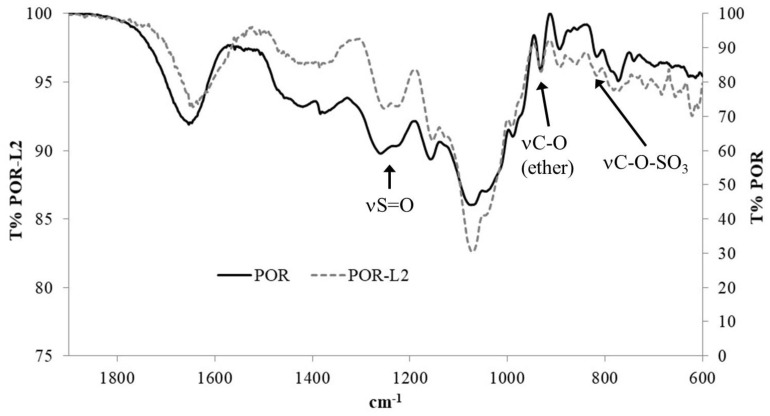
Close-up of the FTIR spectra obtained for **POR** and **POR-L2** in KBr pellet.

Analysis of the porphyrans by FTIR showed sulfate ester S=O stretching bands at ca. 1261 and 1230 cm^−1^ for **POR**, while for **POR-L2** these signals appeared at ca. 1253 and 1226 cm^−1^ (Figure 3, see Figure S5 for the full spectra and Table 1 for selected frequencies). The bands in the 1100–1000 cm^−1^ range were assigned to pyranose ring stretching vibrations. The band at ca. 933 cm^−1^ was attributed to the presence of 3,6-anhydrogalactose (3,6-AG) residues in the polysaccharide structure, while the weak band at ca. 816 cm^−1^ was assigned to primary alkyl sulfate groups [14,15,60,61,62,63]. However, the N-H, aromatic C-H and COO stretching bands could not be observed in POR-L2. The existence of the sulfate signals in **POR-L2** suggests that not all the sulfate moieties may be labile under the reaction conditions used for the immobilization of **L2**.

The bands associated with the O-H and C-H stretching vibrations were also observed respectively at ca. 3416 and 2921 cm^−1^, for **POR** and **POR-L2**.

#### 2.3.2. UV–Vis Analysis

**Figure 4:**
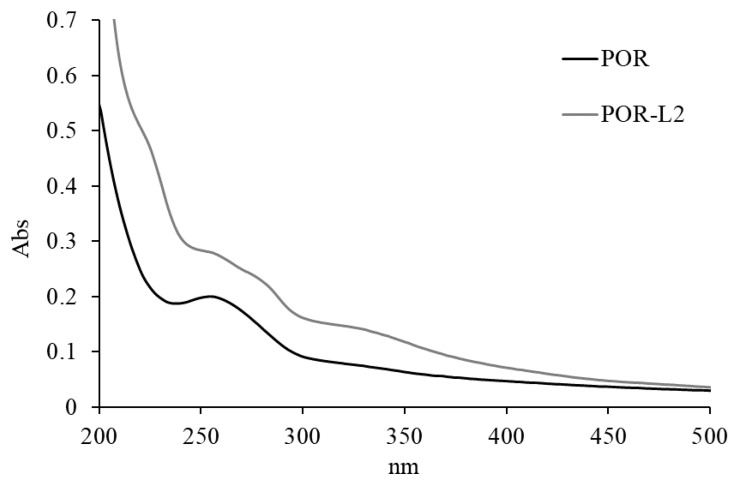
UV–vis absorption spectra of **POR** and **POR-L2** in water, at a concentration of 0.2 mg/mL. The spectra were measured with a 1 cm optical path quartz cell.

To complement the information obtained by FTIR, **POR** and **POR-L2** were analyzed by UV–vis spectroscopy. The native **POR** presented two coalescing absorption bands in the 280–250 nm range, which were assigned to residual proteins in the porphyran structure (Figure 4). In the case of **POR-L2**, the band at ca. 275 nm was more intense and it was possible to observe additional shoulder bands at ca. 330 and 220 nm. The increase of the band at ca. 275 nm and the appearance of the bands at ca. 330 and 220 nm were assigned to aromatic *π-π* electronic transitions of the tyrosine sidechain and 2,4-di-tert-butylphenol moieties, thus suggesting the presence of **L2** units immobilized onto the polysaccharide structure.

#### 2.3.3. NMR Analysis

The measurement of liquid state NMR spectra of hydrocolloids such as carrageenan and porphyran typically requires the adjustment acquisition parameters and/or high temperatures to compensate for the high viscosity of the sample [14,64,65].

**Figure 5:**
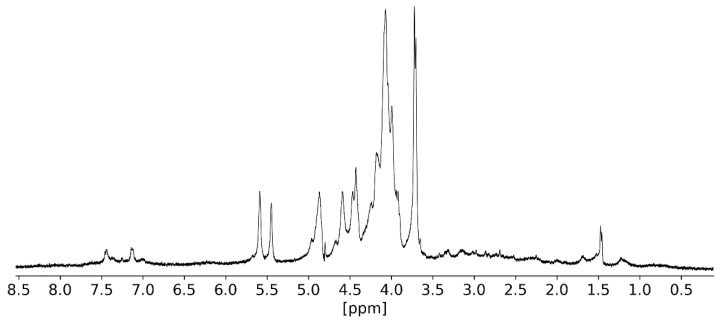
^1^H NMR spectrum of **POR-L2** with suppression of the D_2_O residual signal.

The main objective was to detect the characteristic chemical shifts of the tyrosine aromatic ring within the 7.0–7.6 ppm range. Two main chemical shifts were observed in this range and assigned to the aromatic ring of the tyrosine side chain (Figure 5). The existence of these chemical shifts is consistent with the observations made in the UV–vis studies and further reinforces the indication that **L2** is covalently bonded onto the polysaccharide chain, as intended. Furthermore, it was possible to observe two chemical shifts at ca. 5.57 and 5.45 ppm, which were assigned respectively to the anomeric protons of the 4-O-linked α-l-galactose-6-*O*-sulfate and 3,6-anhydro-α-l-galactose residues [14,15,64,65,66,67].

**Figure 6:**
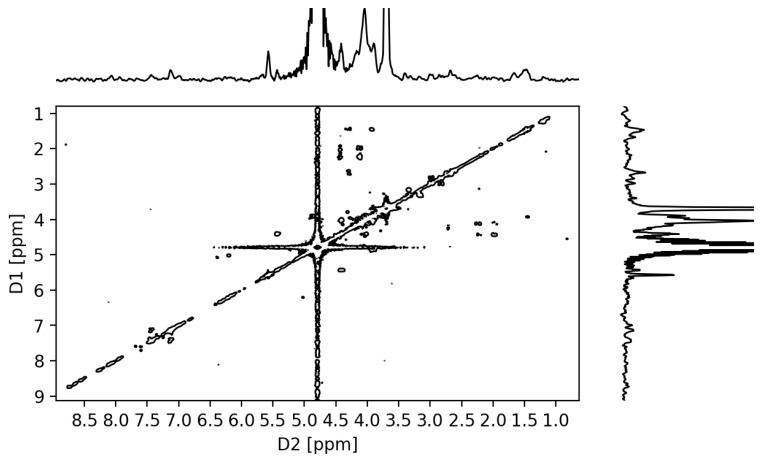
Long-range ^1^H-^1^H COSY spectrum of **POR-L2**, at 50 °C.

The observation of galactose-6-*O*-L2 units was attempted by long-range COSY (Figure 6) and TOCSY experiments (Figure S6), as HMBC experiments yielded very weak signals. The expectation was that the existence of galactose-6-*O*-L2 units would yield long-range couplings between the aromatic protons *ortho* to the sidechain phenolate carbon of tyrosine and the H-6 protons of the galactose-6-*O*-L2 residue. The chemical shifts for the H-6 protons for a putative galactose-6-*O*-tyrosyl residue were expected to be similar to those observed in the literature for the galactose-6-*O*-methyl residues in porphyran, which were reported to be within the 3.5–3.4 ppm range [66,67]. The tyrosine sidechain cross-peaks were observed in the 7.0/7.5 ppm range of the TOCSY spectrum of **POR-L2**, while no cross-peaks were observed in the 8–7/3–4 ppm range. At higher contour levels, the LR-COSY of **POR-L2** presented a weak signal at 3.71/7.45 ppm, but it cannot be unambiguously assigned to the coupling of the tyrosine side-chain aromatic protons with the H-6 protons of the proposed galactose-6-*O*-L2 residues, due to the appearance of additional noise artifacts.

#### 2.3.4. Elemental Analysis

The bulk composition in sulfur and nitrogen of the native and modified porphyrans was obtained by elemental analysis. Native **POR** presented an average%N equal or less than 0.5%, while **POR-L2** presented an average%N of 0.7%. A slight difference in%S was also observed between the native and the modified **POR**, with **POR** and **POR-L2** presenting an average%S of 3.3 and 2.9%, respectively. These results indicate a low degree of sulfate ester substitution in **POR-L2**, as observed in the UV–vis, FTIR, and NMR spectra. This suggests that not all sulfate ester moieties of **POR** are labile under the conditions used for the preparation of **POR-L2**.

### 2.4. Determination of Antioxidant Activity

The native and modified porphyrans were tested for their antioxidant reactivity against [Fe^III^(Phen)_3_]Cl_3_, DPPH, ABTS^•+^, Na[Fe^III^EDTA]/H_2_O_2_/AcOH and Na[Fe^III^EDTA]/O_2_/Benzaldehyde/AcOH. This series of tests allow the screening of the antioxidant activity against strong single-electron oxidants such as [Fe^III^(Phen)_3_]Cl_3_ and radicalar species generated in situ from H_2_O_2_ and benzaldehyde.

#### 2.4.1. Ferric Reducing Activity

Native and modified porphyrans were initially screened for reducing activity in the presence of a strong single-electron oxidant such as [Fe^III^(Phen)_3_]Cl_3_ under acidic conditions. The electronic absorption spectra of the porphyrans in the presence of [Fe^III^(Phen)_3_]Cl_3_ were measured in the visible range after 30 min. The results obtained are presented in and Table 2 (see Figures S7 and S8 for the obtained spectra and Table S1 for the full results).

While the control sample exhibited a small increase of absorption at 510 nm after 30 min, the PORs clearly exhibited some reducing activity after this period at room temperature. Native **POR** presented some reducing activity, and this was attributed to the secondary alcohol groups of the galactose residues in the polysaccharide chain. As expected, **POR-L2** presented better single-electron reducing character than native **POR**. This was attributed to the phenolate functionalities present in the porphyran matrix, which confer the observed activity towards single-electron oxidants.

#### 2.4.2. DPPH and ABTS^•+^ Scavenging Activity

The radical scavenging activity of **POR** and **POR-L2** was tested with modified colorimetric DPPH and ABTS^•^*^+^* assays. The procedures were modified to account for the insolubility of **POR** and **POR-L2** in alcohols and to minimize interferences from these solvents, which may also exhibit some radical-scavenging activity as well [68,69,70,71].

For the DPPH assay, the solvent used was acetonitrile:water (1:1) to ensure that the oxidant and analyte remained in solution. As for the ABTS^•^*^+^* scavenging activity assays, these were carried out in water with a 0.34 mM stock solution of ABTS^•^*^+^*.

The concentration of the tested PORs was 0.2 mg/mL for the DPPH and ABTS^•^*^+^* scavenging activity assays. It was observed that **POR** exhibited very low scavenging activity after 30 min at room temperature, with the scavenged DPPH reaching 6.1% ± 2.9%. A slightly higher scavenging activity was observed with **POR-L2**, reaching 14.8% ± 3.4% of scavenged DPPH after 30 min (Figure S9).

Regarding the ABTS^•*+*^ scavenging activity, **POR** and **POR-L2** presented strikingly different behaviors, with **POR-L2** exhibiting a strong scavenging activity after 1 min at room temperature (Figure S10). **POR** presented a very low ABTS^•*+*^ scavenging activity, with 1.5% ± 0.4% to 2.8% ± 0.9% ABTS^•*+*^ scavenged after 1 min, while **POR-L2** scavenged 40.1% ± 2.1% of ABTS^•*+*^ upon the same time interval. A still more evident indication for the radical scavenging activity of **POR-L2** was observed after 30 min, where % ABTS^•*+*^ scavenged reached 94.2% ± 1.0%, while the native **POR** did not surpass 6.8% ± 1.2%.

This contrast in DPPH and ABTS^•^*^+^* scavenging activities was attributed in part to the different redox potentials of the DPPH/DPPH^−^ and ABTS^•^*^+^*/ABTS couples. The reported redox potentials for the DPPH/DPPH^−^ couple range from 0.16 to 0.33 V vs. SCE, depending on the solvent used, [72] whereas ca. 0.43 V vs. SCE (0.68 V vs. NHE) was reported for the ABTS^•^*^+^*/ABTS couple [73,74,75,76]. Therefore, the stronger radical scavenging activity of **POR-L2** towards ABTS^•^*^+^* may be attributed in part to a stronger oxidising activity of this stable radical compared to DPPH. Nevertheless, **POR-L2** presented a superior radical scavenging activity against DPPH and ABTS^•^*^+^* compared to **POR**. This increased antioxidant activity was attributed to the presence of **L2** units immobilized in the porphyran matrix.

#### 2.4.3. Suppression of the Na[FeEDTA]-Catalysed Oxidation of Methyl Red Dye with H_2_O_2_

The spectroscopic monitoring of the oxidation of methyl red dye by the Na[FeEDTA]/H_2_O_2_/acetic acid system was used to assess the hydroxyl and hydroperoxyl radical scavenging ability of **POR** and **POR-L2** [77].

The change in intensity of the absorption of methyl red at 524 nm in water was monitored after 15 min, which was the time necessary for the red coloration of the dye to become visually indiscernible in the control reactions run without PORs. The control experiments were run without the addition of PORs.

The reaction was carried out in the presence of acetic acid to ensure a controlled reaction between the iron complex and H_2_O_2_ by minimizing the extent of Fenton reactions and enabling the formation of high-valent Fe species, in addition to minimizing the Fe-catalyzed disproportionation of the oxidant and the formation of hydrated iron oxides [78,79,80,81]. The changes in absorbances observed during a 15 min period are listed in Table 3. The obtained spectra are depicted in Figure S11.

It was observed that the PORs exhibited observable suppression behavior after 15 min (Table 3). This was expected: the alcohol moieties of the polysaccharide chain should be capable of exerting some radical scavenging effect, in line with what is reported for simpler alcohols [68,69,70,71]. **POR-L2** presented a noticeably better suppression activity than native **POR**, as was shown by the smaller Δ Abs. This increased suppressing effect was attributed to the radical scavenging activity of the phenolic moieties of **L2** present in the porphyran matrix.

#### 2.4.4. Suppression of the Aerobic Oxidation of Methyl Red Dye with the Na[FeEDTA]/AcOH/Benzaldehyde Oxidant System

The method described in the earlier section was modified by replacing H_2_O_2_ with an aldehyde susceptible to autoxidation. Aldehydes typically oxidize to their corresponding carboxylic acids when in contact with O_2_. This oxidation proceeds through a series of radicalar reactions which involve the formation of a peracid intermediate [82].

In this case, the role of the transition metal complex is to facilitate the autoxidation aldehyde, to bind the peracid intermediate and activate the peracid towards the oxidation of an organic substrate [83].

Thus, the PORs were evaluated for their ability to suppress the process of autoxidation of benzaldehyde, the formation of perbenzoic acid and subsequent degradation of the methyl red dye. The duration of each run was increased to 400 min due the slower kinetics of these oxidation reactions.

The observed changes in absorbance of the PORs are listed in Table 4.

Furthermore, the measured UV-Vis spectra show clear differences between the control and sample runs (Figure S12), demonstrating the antioxidant activity of the tested PORs under these conditions. As mentioned earlier, this antioxidant reactivity stems from the radical scavenging activity typical of alcohols and, in turn, such reactivity should also be observed for these polysaccharides.

A slight difference in activity was also observed between native **POR** and **POR-L2**, with the latter exhibiting only a slightly higher suppression effect in this case. As noted earlier, this could be attributed to the presence of phenolic moieties present in the polymeric chain of the modified porphyrans.

### 2.5. Microstructural Features of Produced Films

The capacity of **POR** and **POR-L2** to form continuous films by mold casting was assessed. **POR**, **POR-L2**, and the equivalent compositions containing 20 wt % glycerol as plasticizer agent (**POR**/gly and **POR-L2**/gly) rendered materials sufficiently strong to resist further manipulation, examination and analysis. Photographs of the obtained films are presented in Figure S13.

**Figure 7:**
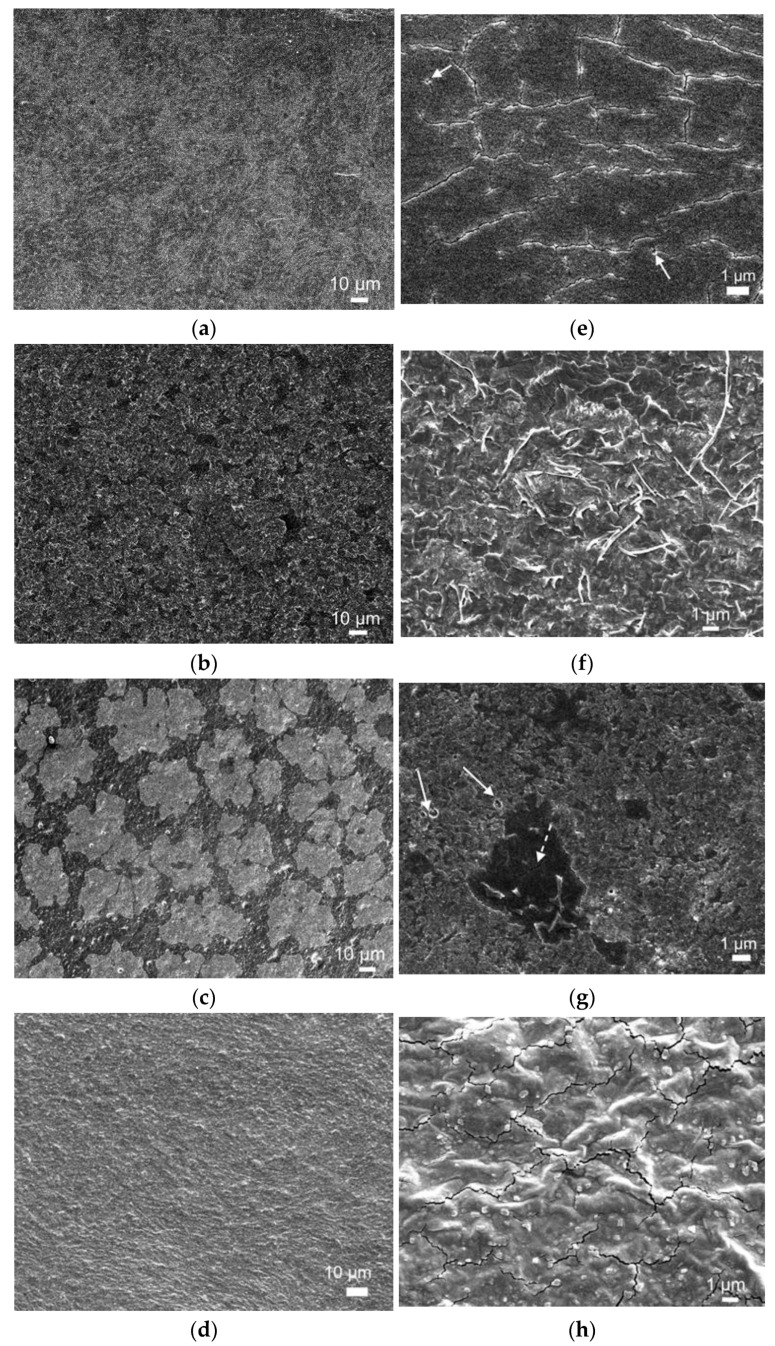
Electron microscope images of the produced films. Low magnification images: (**a**) **POR**; (**b**) **POR**/gly; (**c**) **POR-L2**; (**d**) **POR-L2**/gly. High magnification images: (**e**) **POR** (arrows highlight pores); (**f**) **POR**/gly; (**g**) **POR-L2** (arrows highlight pores, a crack is indicated with dashed arrow); (**h**) **POR-L2**/gly.

Electron microscope images show that all produced films are continuous (Figure 7a–d); high magnification images (Figure 7e–h) enlighten different microstructural features depending on composition.

**POR** films are smooth and continuous (Figure 7a) although not fully dense, with microcracks and pores visible at high magnification (Figure 7e, arrow). Addition of glycerol (gly) plasticizer strongly changes film microstructure: **POR**-gly displays higher surface roughness (Figure 7c) and development of flaky features (Figure 7d), in good agreement with results by other authors [84]. Film density also appears to increase, with less visible cracks and pores. EDS elemental analysis (Table 5) showed that **POR** and **POR**-gly films are mainly composed of C, O and S; residual amounts of Mg, Na, K and Ca were also detected. Although Figure 7a suggests the presence of a biphasic mixture in **POR**, EDS compositional difference between the darker and lighter regions is not statistically significant.

**POR-L2** films are continuous, smooth and dense (Figure 7c), although some pores are visible (Figure 7g). Average film composition is displayed in Table 5. The microstructure is clearly biphasic and EDS point analysis shows that the main difference regards the concentration of S (2.54 ± 0.06 and 1.07 ± 0.78 at%, in the lighter and darker phase respectively) and K (2.71 ± 0.31 at% in the lighter phase, not detected in the darker phase). Addition of glycerin (**POR-L2**/gly) again appears to increase film roughness compared to the system without plasticizer (**POR-L2**), as well as to improve compositional uniformity (Figure 7c). A second phase is still visible in the form of small cubic crystals with side around 100 nm (Figure 7h), but no statistically significant difference was found between their composition and the surrounding matrix. Similarly to **POR**, this suggest that the difference between phases refers to crystallinity degree. Notably, both **POR** (Figure 7e) and **POR-L2**/gly (Figure 7h) films display microcracking pattern typical of differential contraction on drying. This is probably due to capillary shrinkage at the evaporation surface limited by adhesion to the Petri dish on the bottom surface, resulting in sufficiently high tensile stress to compete with material cohesion [85]. Further work regarding these issues and their relation with the films’ mechanical properties is ongoing.

### 2.6. Antimicrobial Activity

The inhibition of bacterial growth upon exposure to the porphyran film solutions is presented in Table 6. The solutions displayed noticeable potential in the inhibition of *S. aureus*, with the lowest bacterial growth observed in the highest concentration of **POR-L2** tested (1.6 mg/mL). Exposure to a different batch of **POR-L2** led to lower, yet still above 50% inhibition of the gram-positive strain at 1.6 mg/mL. No significant inhibition of the gram-negative *E. coli* was observed in all conditions tested. The apparent specificity of antimicrobial action in the film solutions, limited to gram-positive bacteria, is a promising indicator of their potential as preservative agents for food packaging or as an antimicrobial cutaneous dressing. The results also reinforce an identical mechanism of antimicrobial action between the formulations tested, as both solutions limited their activity to the Gram-positive strain, with only different magnitudes of inhibition between them. Growth reductions of less than 10% seen on the pure porphyran solution indicate that the activities verified on the modified samples are most likely due to the presence of the substituted phenol.

While the antimicrobial activity of 2,4-di-tert-butylphenol against gram-positive bacteria is widely reported, limited data is available on the inhibition of gram-negative bacteria by the same compound. While there are several studies dealing with inhibition of growth of Gram-negative *Pseudomonas aeruginosa* and *Serratia marcescens* by 2,4-di-tert-butylphenol [36,86,87,88] no information regarding *E. coli* inhibition appears to have been published. Govender et al. (2016) tested the in-vitro antimicrobial capabilities of a large selection of lipophilic derivatives of synthesized phenols and naphthols. Mirroring the results obtained in our study, these authors reported microbial inhibition almost exclusively over the Gram-positive strain *S. aureus*, and discouraged the possibility that a non-specific membrane-altering activity was behind the antimicrobial potential of lipophilic phenols. Instead, inhibition of serine proteases and β-lactamases due to the metabolism of quinone methides was pointed as the likely cause of this specificity [89].

2,4-di-tert-butylphenol has also been associated with antifungal activity, with in-vitro concentrations of 25 μg/mL achieving near complete inhibition of *Candida albicans* [90]. Anti-biofilm assays and morphological observation led the authors to assume that an inhibition of hyphal development was the cause of this anti-fungal activity, but complementary research is yet to be performed.

The mechanisms of inhibition could not be verified in the antimicrobial assays conducted in this study, but are an important indicator of other means by which the films produced may yield antimicrobial activity in different situations. Immediate further testing of these films will attempt to diversify the organisms in an attempt to confirm their antibacterial specificity and the possibility of fungal growth inhibition. Considering the reported cytotoxicity of 2,4-di-tert-butylphenol on HeLa, MCF-7, and A431 cell lines, an additional screening of the effect of the modified film on melanocyte and keratinocyte cell lines might unlock a new venue for their application as vehicle for skin therapeutical compounds [39,91,92];). Additionally, cytotoxicity on 3T3 cell lines could provide useful information on the safety of the active agent at the concentrations present in the porphyran films for food applications.

## 3. Materials and Methods

Powdered *Porphyra dioica* was purchased from ALGAPlus (Ílhavo, Portugal). L-Tyrosine, 2,4-di-tert-butylphenol, benzaldehyde, 1,10-phenantroline (Phen), 2,2-diphenyl-1-picrylhydrazyl (DPPH) and Mueller-Hinton broth 2 were from Sigma-Aldrich (St. Louis, MO, USA). Sodium hydroxide was from EKA (Bohus, Sweden). Glycerol (86–88%) was from Scharlau Chemie S.A. (Sentmenat, Spain), Paraformaldehyde and Fe^III^Cl_3_.6H_2_O were from Panreac Química SLU (Barcelona, Spain). Glacial Acetic acid and HCl (37% solution) were from VWR Chemicals (Fontenay-sous-Bois, France). Disodium EDTA was from VWR Chemicals (Leuven, Belgium). Methyl red was from Merck KGaA (Darmstadt, Germany). Hydrogen peroxide 30% solution (phosphate stabilized) was from Chem-Lab NV (Zedelgem, Belgium) and diammonium 2,2′-azino-bis(3-ethylbenzthiazoline-6-sulfonate) (ABTS) and ammonium persulfate were from Alfa Aesar (Kandel, Germany). Solvents were purchased from Sigma-Aldrich, Chem-Lab, and Carlo-Erba Reagents S.A.S. (Val de Reuil, France). The reagents and solvents were used as received. Ferric Sodium N,N,N′,N′,-ethylenediaminetetraacetate (Na[FeEDTA]) was prepared using methods reported in the literature [93].

Electronic absorption spectra (UV–vis) spectra were recorded with a Thermo Scientific 201 spectrophotometer (Thermo Fisher Scientific Inc., Waltham, MA, USA) using Insight 2 software v2.1.175. All samples for UV–vis analysis were prepared and measured in triplicate.

The ^1^H and ^13^C NMR spectra were measured on Bruker Avance II+ 400 MHz and 300 MHz Spectrometers (Bruker BioSpin AG, Fällanden, Switzerland). Further spectra processing was carried out with Ssnake v1.3 and rNMR v1.1.9 package for R software v 4.0.3 [94,95,96]. ^1^H and ^13^C chemical shifts (δ) are expressed in ppm relative to the deuterated solvent residual peak. The sample for NMR analysis of POR-L2 was prepared by dissolving 12 mg of the polysaccharide in 0.6 mL of D_2_O. The ^1^H NMR spectra for POR-L2 were obtained at 50 °C, with eight scans and solvent suppression [97]. The LR-COSY spectra of POR-L2 were obtained with the *cosylrqf* pulse program at 50 °C, with 60 scans per cycle [98]. The TOCSY spectrum was obtained with the *mlevetgp* pulse grogram at 50 °C, with 48 scans per cycle [99].

Elemental analysis was carried out at Laboratório de Análises of Instituto Superior Técnico, using an EA1108 CHNS-O elemental analyzer by Carlo Erba Instruments (represented by Fison Instruments Ltd., Glasgow, UK). Transmittance FT-IR spectra were recorded in the 4000–600 cm^−1^ range with a JASCO FT/IR-430 spectrometer (JASCO International Co. Ltd., Hachioji, Tokyo, Japan), using samples dispersed in KBr pellets.

The procedures for the extraction of porphyran, the synthesis of modified L-tyrosines, the chemical modification of porphyran and preparation of porphyran films are included in the supporting material. The porphyran films were prepared according to reported methods, with adaptations [100].

The ferric reducing power assay was carried out adapting a method reported in the literature [101]. The 2,2-diphenyl-1-picrylhydrazyl (DPPH) and ABTS^•+^ radical scavenging activity assays were performed adapting a method from the literature [102,103,104]. The adapted procedures for the ferric reducing power, DPPH, ABTS^•+^, Na[FeEDTA]/AcOH/H_2_O_2_ and Na[FeEDTA]/AcOH/benzaldehyde and antimicrobial assays are described in the supporting material.

## 4. Conclusions

Porphyran was extracted from commercial powdered *Porphyra dioica* and treated with a L-tyrosine derivative (**L2**), under alkaline conditions, to achieve the direct modification of the polysaccharide chain without resorting to additional activating agents. The results indicate that **POR-L2** presents marked increase in antioxidant activity and some increased antimicrobial when compared to the native **POR**. The reasons for the increase of these activities are attributed to the existence of **L2** units on the polysaccharide chain.

The results obtained from UV–vis, NMR and the antioxidant assays suggest that **L2** was covalently grafted to reasonable extent onto the polysaccharide chain following a S_N_2 reaction between the galactose-6-*O*-sulfate units in porphyran and **L2**. However, the LR-COSY NMR experiments did not yield unambiguous data regarding the existence of galactose-6-*O*-**L2** residues.

**POR** and **POR-L2** can form smooth continuous films by simple casting and drying on a polystyrene mold. The inclusion of glycerol as plasticizer resulted in improvements on overall film continuity by reducing fissures and pores. Our data show that native **POR** and **POR-L2** can serve, for instance, as protective films. The optimization of the mechanical characteristics of the films formed with porphyran is under way.

The porphyran treated with **L2**-**POR-L2**-presented the added advantage of increased antioxidant and antimicrobial activity compared to **POR**. In turn, **POR-L2** may serve as a starting point for the development of protective, washable films for perishable foods, thus may be of relevance to produce films and coatings for food packaging with improved protecting characteristics.

## Figures and Tables

**Table 1:** Selected IR bands (in cm^−1^) for **POR** and **POR-L2**.

Compound	υ(O-H)	υ(C-H)	υ(S=O)	υ(C-O, ether)	υ(C-O-SO_3_)
**POR**	3444.7 (*b*)	2929.3 (*w*)	1259.8 (*m*) 1230.4 (*sh*)	931.9 (*w*)	815.5 (*w*)
**POR-L2**	3418.2 (*b*)	2932.2 (*w*)	1252.1 (*m*) 1225.5 (*sh*)	931.4 (*w*)	817.2 (*w*)

*b*: broad, *sh*: shoulder, *w*: weak, *m*: medium, *s*: strong; *vs*: very strong.

**Table 2:** Obtained absorbances for the tested porphyrans after 30 min in the ferric reduction activity screening

Sample ^a^	t (min)	Abs 510 nm ^b^
Control ^c^	0	0.042 ± 0.003
	30	0.045 ± 0.002
**POR** ^d^	30	0.108 ± 0.002
**POR-L2** ^d^	30	0.161 ± 0.002

^a^ Concentration of PORs in the samples was 0.2 mg/mL and [Fe^III^(Phen)_3_]Cl_3_ was 0.55 mM. Experiments were performed in triplicate using a quartz cell with a 1 cm optical path. ^b^ The obtained values are presented as mean ± SD. ^c^ The control sample was [Fe^III^(Phen)_3_]Cl_3_ in water at 0.55 mM (1.65 × 10^−6^ mol, 3 mL), in the absence of porphyrans. ^d^ The presented absorbances were obtained for after subtraction with the measured absorbances at 510 nm for **POR** and **POR-L2** at 0.2 mg/mL, in water, as described in Section 2.3 (Table S1).

**Table 3:** Observed absorbance changes obtained for the tested porphyrans

Sample ^a^	Δ Abs 524 nm ^b^
Control	0.0886 ± 0.0043
**POR**	0.0663 ± 0.0018
**POR-L2**	0.0464 ± 0.0031

^a^ Concentration of the porphyran samples was 0.2 mg/mL. ^b^ Experiments were performed in triplicate and the results are presented as mean ± SD.

**Table 4:** Observed absorbance changes obtained for the tested porphyrans

Sample ^a^	Δ Abs 500 nm ^b^
Control ^c^	0.0351 ± 0.0034
**POR**	0.0065 ± 0.0031
**POR-L2**	0.0012 ± 0.0010

^a^ Concentration of the porphyran samples was 0.16 mg/mL. ^b^ Experiments were performed in triplicate and the results are presented as mean ± SD. ^c^ The control was carried out with distilled water instead of the porphyran sample.

**Table 5:** Average elemental composition of produced films determined by EDS area analysis.

	POR	POR/gly	POR-L2	POR-L2/gly
C (at%)	59.2 ± 1.1	45.8 ± 1.2	57.8 ± 2.1	46.9 ± 5.8
O (at%)	38.4 ± 1.6	46.5 ± 3.3	37.8 ± 2.8	39.4 ± 4.9
S (at%)	1.4 ± 0.3	3.3 ± 0.2	1.0 ± 0.1	4.3 ± 0.7
Mg (at%)	0.2 ± 0.1	0.5 ± 0.1	0.1 ± 0.0	0.1 ± 0.0
Na (at%)	0.4 ± 0.1	0.7 ± 0.2	0.4 ± 0.1	1.4 ± 0.1
K (at%)	*nd*	0.9 ± 0.3	2.9 ± 0.7	7.6 ± 2.2
Ca (at%)	*nd*	0.6 ± 0.2	*nd*	0.3 ± 0.1

*nd*: not detected.

**Table 6:** Percentual reduction of microbial growth for strains of *S. aureus* and *E.coli* under exposure to porphyran and modified porphyran film solutions. Results presented are the mean ± std. deviation of three independent assays, in which four technical replicas were conducted. nd = not detectable.

Solution Concentration (mg/mL)	% Growth Inhibition	
	*S. aureus*	*E. coli*
**POR**		
0.2	*nd*	*nd*
0.4	*nd*	*nd*
0.8	4.9 ± 0.7	*nd*
1.6	6.6 ± 0.4	*nd*
**POR-L2**		
0.2	*nd*	*nd*
0.4	*nd*	*nd*
0.8	27.3 ± 5.8	3.1 ±2.5
1.6	56.3 ± 3.0	4.5 ± 2.62
**POR-L2**		
0.2	*nd*	*nd*.
0.4	18.9 ± 2.1	*nd*
0.8	43.3 ± 4.7	*nd*
1.6	65.7 ± 2.1	*nd*

## Supplementary Materials

The following are available online: Scheme S1. General reaction outline for the synthesis of **L2**; Figure S1. ^1^H NMR spectrum of **L1** in Acetone-D_6_/HCl. The chemical shifts at ca. 2 and 6 ppm correspond to the solvent and HCl peaks, respectively; Figure S2. ^1^H NMR spectrum of **L2** in acetone-D_6_/HCl. The chemical shifts at ca. 2 and 6.3 ppm correspond to the solvent and HCl peaks, respectively; Figure S3. ^13^C APT spectrum of **L2** in acetone-D_6_/HCl. The chemical shift at ca. 30 ppm corresponds to the solvent residual peak; Figure S4. HMBC spectrum of **L2** in acetone-D_6_/HCl; Scheme S2. General outline of the S_N_2 alkylation reaction between the galactose-6-*O*-sulfate units in porphyran and **L2**, in the presence of OH^−^; Scheme S3. General outline of the intramolecular S_N_2 cyclisation reaction of the galactose-6-*O*-sulfate units in porphyran; Figure S5. FTIR spectra of **POR** and **POR-L2** in a KBr pellet; Figure S6. ^1^H-^1^H TOCSY spectrum of **POR-L2**; Figure S7—Electronic absorption spectra of the ferric reducing activity assays after 30 min. The control sample was [Fe^III^(Phen)_3_]Cl_3_ in water at 0.55 mM (1.65 × 10^−6^ mol, 3 mL), in the absence of porphyrans. The concentration of the porphyran samples (**POR** and **POR-L2**) was 0.2 mg/mL; Figure S8. Electronic absorption spectra of ferric reducing activity assays carried out with **POR** and **POR-L2,** after subtraction of the respective spectra at 0.2 mg, without 0.55 mM [Fe^III^(Phen)_3_]Cl_3_; Table S1. Obtained absorbances for the tested porphyrans after 30 min in the ferric reduction activity screening; Figure S9. Electronic absorption spectra of the DPPH^•^ radical in water and in the presence of either ascorbic acid, **POR** or **POR-L2**, after 30 min. The mass concentration of the PORs was 0.2 mg/mL. The spectra were measured with 1 cm optical path quartz cells; Figure S10. Electronic absorption spectra of the ABTS^•^*^+^* radical cation in water and in the presence of either **POR** or **POR-L2**. The mass concentration of the PORs was 0.2 mg/mL. The spectra were measured with a 1 cm optical path quartz cell; Figure S11. Electronic absorption spectra obtained for the *Na[FeEDTA]/AcOH/H_2_O_2_* assays: control run (A), **POR** (B) and **POR-L2** (C) after 15 min under the described oxidation conditions, at a mass concentration of 0.2 mg/mL in water. The spectra were measured with 1 cm optical path quartz cells; Figure S12. Electronic absorption spectra obtained for the *Na[FeEDTA]/AcOH/Benzaldehyde* assays, after 400 min under the described aerobic oxidation conditions, at a mass concentration of 0.16 mg/mL in acetonitrile/water (3:2). The control run was carried out with distilled water instead of the porphyran sample. The spectra were measured with a 1 cm optical path quartz cell; Figure S13. Macrographs of the produced films: (a) POR; (b) POR/gly; (c) POR-L2.; (d) POR-L2/gly.

## Sample Availability

Samples of the compounds are not available from the authors.
