# Supplementary material for: Enhancement of the Antioxidant and Antimicrobial Activities of Porphyran through Chemical Modification with Tyrosine Derivatives
Source: Molecules. 2021 May 14;26(10):2916. doi: 10.3390/molecules26102916 (PMC8156949; doi:10.3390/molecules26102916)
Supplement: Supplementary file 1 [file molecules-26-02916-s001.zip › molecules-1205807-supplementary.pdf]

Enhancement of the antioxidant and antimicrobial activities of porphyran through chemical modification with tyrosine derivatives

Pedro Adão,[a]* João Reboleira,[a] Marco Teles,[a] Beatriz Santos,[a] Nádia Ribeiro,[c] Carlos M. Teixeira,[c] Mafalda Guedes,[d,e] João Costa Pessoa,[c] Susana Bernardino[b]

[a] *MARE, Politécnico de Leiria, Portugal*

[b]*MARE, Escola Superior de Turismo e Tecnologia do Mar, Politécnico de Leiria, Portugal*

[c]*Centro Química Estrutural, Departamento de Engenharia Química, Instituto Superior Técnico, ULisboa, Av. Rovísco Pais, 1049-001 Lisboa, Portugal*

[d] *CDP2T and Dep. Mechanical Engineering, Escola Superior de Tecnologia de Setúbal, Instituto Politécnico de Setúbal, 2910-761 Setúbal, Portugal.*

[e] *CeFEMA, Instituto Superior Técnico, ULisboa, Av. Rovisco Pais, 1049-001 Lisboa, Portugal*

Supporting Material

Page

1. Extraction procedure of porphyran from Porphyra dioica………………………………………. 1
2. Synthesis of L-tyrosine derivatives……………………………………………………………… 2
3. Chemical modification of porphyran……………………………………………………………. 6
4. Ferric reducing power assay with $[Fe^{III}(Phen)_3]Cl_3$………………………………………….. 7
5. DPPH and $ABTS^{\bullet+}$ *scavenging activity assays*……………………………………………. 7
6. Suppression of the Na[FeEDTA]-catalysed oxidation of methyl red dye with H_2O_2………….. 8
7.Suppression of the aerobic oxidation of methyl red dye with Na[FeEDTA]/AcOH/Benzaldehyde oxidant system……………………………………………. 9
8. Preparation of POR and POR-L2 films…………………………………………………………. 9
9. Antimicrobiological activity assays ………………………………………………………………. 10
10. FTIR and NMR of POR-L2…………………………………………………………………….. 10
11. UV-Vis spectra of the ferric reducing power assays……………………………………………. 12
12. UV-Vis spectra of the DPPH and ABTS•+ assays……………………………………………… 14
13. UV-Vis spectra of the Na[FeEDTA]/AcOH/H2O2 and Na[FeEDTA]/AcOH/Benzaldehyde assays…………………………………………………………………………………………….. 15
14. Macrographs of the porphyran films…………………………………………………………... 17

1. Extraction procedure of porphyran from Porphyra dioica

Powdered *Porphyra dioica* (5 g) was evenly spread over half of a 29.5×23.0 cm sheet of industrial cleaning paper. The paper was carefully folded in half and rolled. The resulting paper roll was moistened with water and placed in a 125 mL soxhlet extractor. Two paper rolls were used for an extraction run. Water (500 mL) was charged into a 1 L round-

bottom flask, along with ceramic chips. The extractor and a reflux condenser were mounted onto the flask and the extraction liquid was gently heated to the boiling point. The temperature was carefully controlled to minimise the amount of foaming produced by the extracted porphyran. The extraction was carried out for a total of 8 h, till a clear amber liquid was obtained. The liquid was filtered hot with a quantitative paper filter to remove solid impurities. The liquid was transferred to a 1 L round-bottom flask and was carefully evaporated in a rotary evaporator till a highly viscous residue was obtained. The polysaccharide was precipitated with the addition of ca. 500 mL of isopropyl alcohol to the viscous residue. The resulting suspension was stirred vigorously and allowed to rest. The supernatant liquid was carefully decanted, and ca. 300 mL of isopropyl alcohol were added to the precipitate. The suspension was transferred to a blender and triturated for ca. 5 minutes. The precipitate was filtered with a quantitative filter paper and washed with isopropyl alcohol. The cream-coloured solid was oven-dried at 50 ºC for 72 h. Yields obtained from various extraction runs ranged from 1.6 to 2.4 g. FTIR (ν_{max}/cm^{-1}): 1259, 1230 (sulfate, m), 816 (primary alkylsulfate, w). Elemental Analysis: **Found**: %C, 34.85; %H, 5.17; %N, 0.51%; %S, 3.32 %.

2. Synthesis of L-tyrosine derivatives

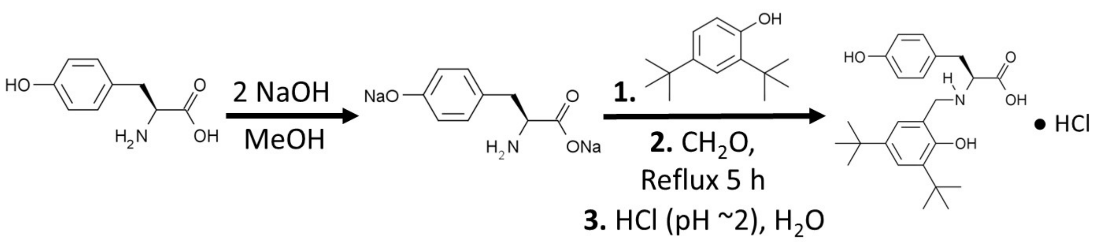

Scheme SI1. General reaction outline for the synthesis of **L2**.

L1 – L-tyrosine (20 mmol) was dissolved in methanol (150 mL) with NaOH (40 mmol) at room temperature. Thymol (20 mmol) was added to the mixture upon complete dissolution of the amino acid. After the complete dissolution of the phenol, paraformaldehyde (20 mmol) was added to the reaction mixture. The resulting mixture was heated to reflux with stirring for 5 h, after which the mixture was allowed to cool to room temperature. The pH of the reaction mixture was adjusted to *ca.* 3 with dilute HCl, followed by the addition of 250 mL of water to induce the precipitation of the product.

The suspension was stirred at room temperature for ca. 30 min. and the precipitate was filtered and washed with water and diethyl ether. Yield: 0.9 g. **Analysis Calculated** for $C_{20}H_{25}NO_4$•0.5 H_2O: %C, 68.18; %H, 7.44; %N, 3.97; **Found**: %C, 67.69; %H, 7.47; %N, 3.93. The ^{1}H-NMR and ^{13}C-{^{1}H} NMR spectra measured in acetone-D_6/HCl indicated that a mixture of compounds was obtained. As a result, it was not possible unambiguously assign the observed chemical shifts.

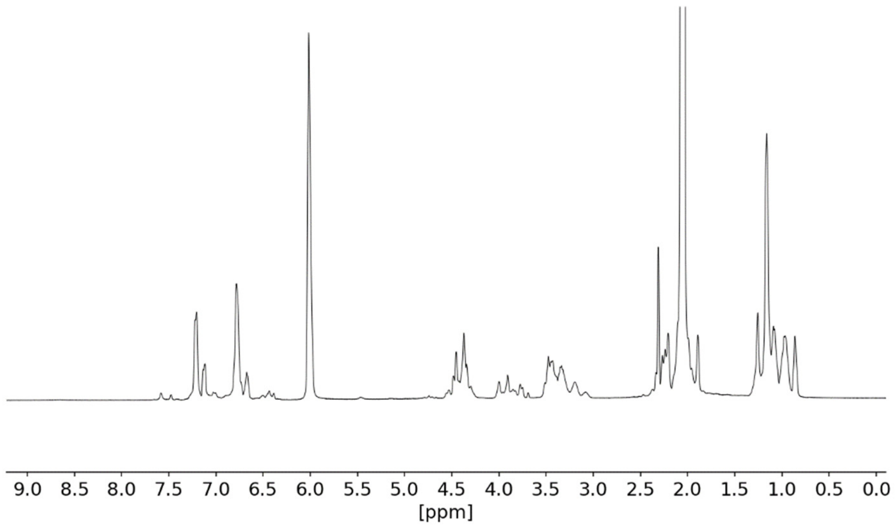

Figure SI1. ^{1}H NMR spectrum of **L1** in Acetone-D_6/HCl. The chemical shifts at ca. 2 and 6 ppm correspond to the solvent and HCl peaks, respectively.

L2 – L-tyrosine (20 mmol) was dissolved in methanol (150 mL) with NaOH (40 mmol) at room temperature. 2,4-di-tert-butylphenol (20 mmol) was added to the mixture after complete dissolution of the amino acid. After complete dissolution of the phenol, paraformaldehyde (20 mmol) was added to the reaction mixture. The resulting mixture was heated to reflux with stirring for 5 h, after which the mixture was cooled to room temperature. The pH of the reaction mixture was adjusted to *ca*. 2 with dilute HCl followed by the addition of 600 mL of water to induce the precipitation of the product. The suspension was stirred at room temperature for ca. 30 min. and the precipitate was filtered under vacuum and washed with water. The obtained white precipitate was recovered and suspended in ca. 100 mL diethyl ether. The resulting suspension was stirred for at least 10 min and the resulting precipitate was filtered under vacuum washed with diethyl ether. The white solid was recovered, and oven dried at 50 ºC. Yield: 2.4 g. **Analysis Calculated** for $C_{24}H_{33}NO_4$•H_2O: %C, 69.04; %H, 8.45; %N, 3.35; **Found**: %C,

69.41; %H, 8.36; %N, 3.60. ^{1}H-NMR (300 MHz, acetone-D_6/HCl, ppm): δ 1.23, 1.39 [18 H, s, $(C\underline{H}_3)_3CC_{aryl}$], 3.44, 3.46 [2 H, m, $C_{aryl}C\underline{H_2}CH$, side-chain], 4.31 [1 H, m, $C_{aryl}CH_2C\underline{H}$], 4.29, 4.33, 4.38, 4.42 [2 H, m, $C_{aryl}C\underline{H}_2NH_2^+$], 6.73, 6.76, 7.19, 7.22 [4 H, aromatic, side-chain], 7.21, 7.36 [2 H, aromatic]; ^{13}C-{^{1}H} NMR (75 MHz, acetone-D_6/HCl, ppm): δ 30.40, 31.67 [6 C, $(\underline{C}H_3)_3CC_{aryl}$], 34.70, 35.84 [2 C, $(CH_3)_3\underline{C}C_{aryl}$], 35.08 [1 C, $C_{aryl}\underline{C}H_2CH$, side-chain], 47.94 [1 C, $C_{aryl}\underline{C}H_2NH_2^+$], 61.27 [1 C, $C_{aryl}CH_2\underline{C}H$], 116.32, 121.48, 124.09, 125.51, 126.21, 128.41, 141.20, 144.04, 153.20, 157.77 [12 C, aromatic, side-chain], 169.99 [1 C, carboxylate];

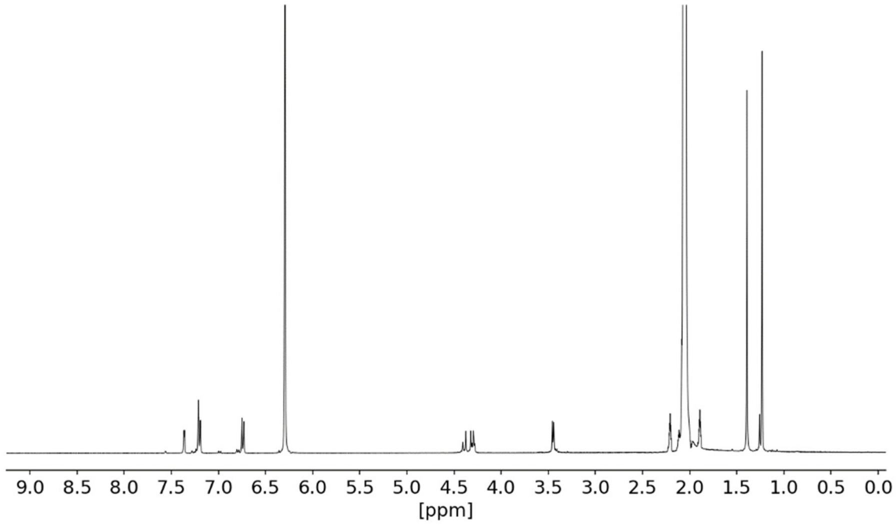

Figure SI2. ^{1}H NMR spectrum of **L2** in acetone-D_6/HCl. The chemical shifts at ca. 2 and 6.3 ppm correspond to the solvent and HCl peaks, respectively.

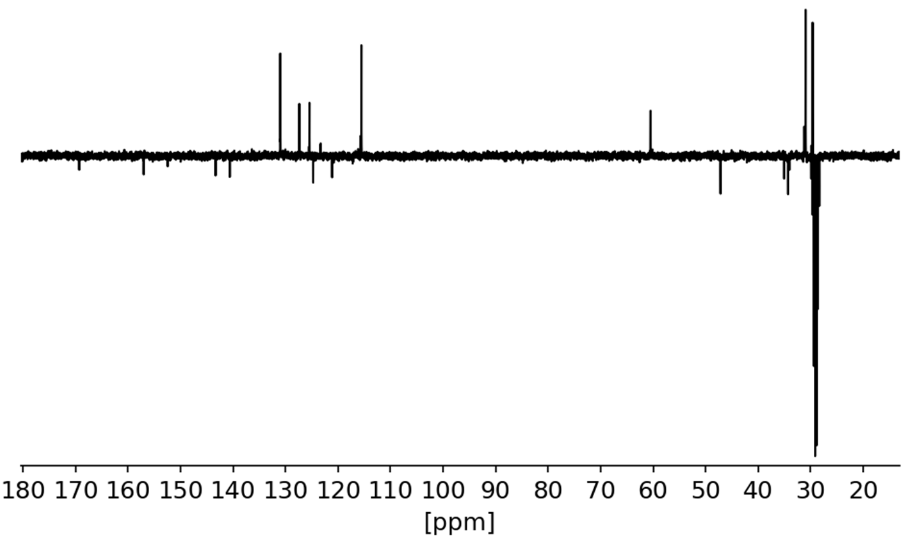

Figure SI3. ^{13}C APT spectrum of **L2** in acetone-D_6/HCl. The chemical shift at ca. 30 ppm corresponds to the solvent residual peak.

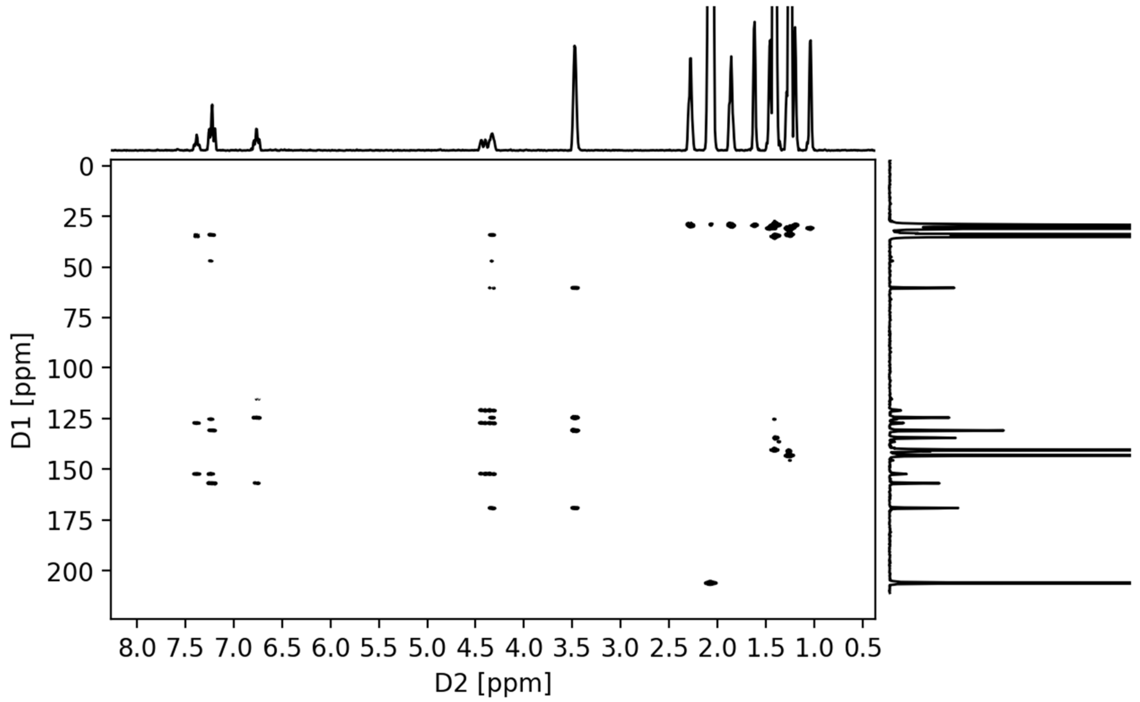

Figure SI4. HMBC spectrum of **L2** in acetone-D_6/HCl.

3. Chemical modification of porphyran

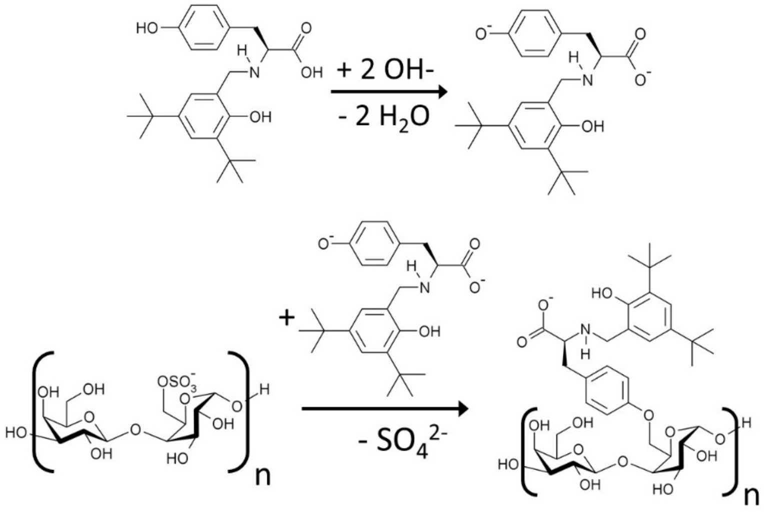

Scheme SI2. General outline of the S_N2 alkylation reaction between the galactose-6-*O*-sulfate units in porphyran and **L2**, in the presence of OH^-.

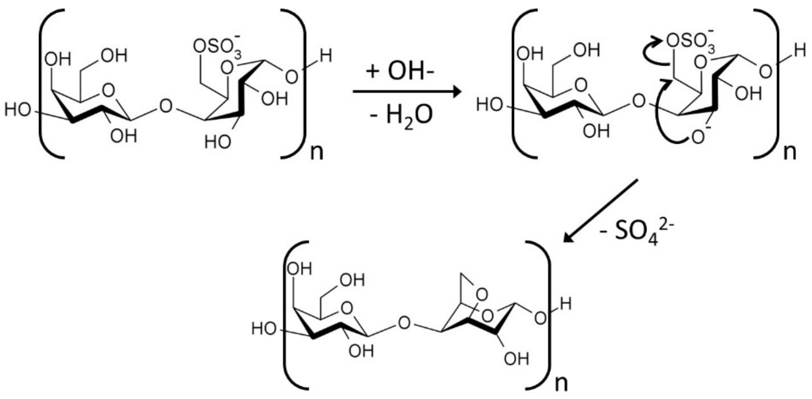

Scheme SI3. General outline of the intramolecular S_N2 cyclisation reaction of the galactose-6-*O*-sulfate units in porphyran.

POR-L2 – In a round-bottom flask, porphyran (2.9 g) was dissolved in 150 mL of water and the suspension was heated to 70 ºC with stirring. After the dissolution of the porphyran, 150 mL of isopropyl alcohol was added to the mixture, followed by **L2** (1.5 g, 3.7 mmol) and KOH (0.5 g, 7.4 mmol). The round-bottom-flask was equipped with a reflux condenser and the mixture was stirred 70 ºC for 24 h. After that period, the solvent was evaporated till a highly viscous residue was obtained. This residue was precipitated with 500 mL of isopropyl alcohol and the liquid was carefully decanted. Additional 300

mL isopropyl alcohol was added to the precipitate and the suspension was triturated in a blender. The precipitate was filtered under vacuum with a quantitative filter paper and washed thoroughly with isopropyl alcohol and acetone. The light brown solid was recovered (1.9 g) and oven-dried at 50 ºC for 72 h. FTIR (ν_{max}/cm^{-1}): 1252, 1225 (sulfate, m), 817 (primary alkylsulfate, w). Elemental Analysis: **Found**: %C, 34.25; %H, 4.80; %N, 0.71; %S, 2.90 %.

4. Ferric reducing power assay with [Fe^{III}(Phen)$_3$]Cl3

A stock solution of 3.3 mM [Fe^{III}(Phen)$_3$]Cl$_3$ was prepared by carefully dissolving Fe^{III}Cl$_3$.6H$_2$O (89.1 mg, 3.3×10^{-4} mol), concentrated HCl (165.0 μL) and 1,10-phenanthroline monohydrate (198.0 mg , 1.0×10^{-3} mol) in 100.0 mL of distilled water, in this order.

The preparation of the assay runs was as follows: A 15 mL conical tube was charged with 1.5 mL of water, 1 mL of stock solution of the porphyran sample at 0.6 mg/mL and 0.5 mL of a 3.3 mM solution of [Fe^{III}(Phen)$_3$]Cl$_3$. The tube was stoppered, the mixture was stirred and allowed to rest for 30 min in the dark, at room temperature. After this period, the electronic absorption spectrum was measured from 650 to 400 nm, using a quartz cell with an optical path of 1 cm.

5. DPPH and ABTS$^{•+}$ scavenging activity assays

The 2,2-diphenyl-1-picrylhydrazyl (DPPH) and ABTS$^{•+}$ radical scavenging activity assays were performed adapting a method from the literature [[1],[2],[3]]. A stock solution of DPPH (5 mg) in acetonitrile (50 mL) was prepared for immediate use. The final concentration the DPPH stock solution was 0.1 mg/mL (0.2536 mM). A stock solution of the porphyran was prepared by dissolving 15 mg of polysaccharide in 25 mL of phosphate buffer at pH 5.5 (80 mM), at 70 ºC.

The scavenging reaction was carried out by mixing 1 mL of DPPH stock, 0.5 mL acetonitrile, 0.5 mL phosphate buffer and 1 mL of porphyran stock at 0.6 mg/mL in a glass test tube, and allowing the mixture to stand for 30 min in the dark. The UV-Vis spectrum of the sample was then measured in the 700-400 nm range.

The stock solution of $ABTS^{•+}$ was prepared by dissolving 47.3 mg of ABTS and 13.0 mg of potassium persulfate in 250 mL of distilled water. The mixture was shaken till the complete dissolution of the reagents and was left to stand in the dark for 24 h prior use. The final concentration of $ABTS^{•+}$ in the stock solution was 0.1892 mg/mL (0.3448 mM). The stock solutions of the porphyrans at 0.6 mg/mL were prepared by dissolving 15 mg of polysaccharide in 25 mL of distilled water, at 70 ºC.

The scavenging reaction was carried out by mixing 2 mL of $ABTS^{•+}$ stock, 2 mL of distilled water and 2 mL of porphyran stock in a glass test tube. The mixture was left to stand for 1 min in the dark. The UV-Vis spectra of the sample were measured after 1 min and after 30 min, in the 700-400 nm range. The final concentration of porphyran in the test tubes was 0.2 mg/mL in both the DPPH and $ABTS^{•+}$ assays. The observed scavenging activity in both assays was calculated using the following expression:

$$\% \text{ radical scavenged} = \left(\frac{A_{control} - A_{sample}}{A_{control}}\right) \times 100$$

Where $A_{control}$ and A_{sample} correspond to the control and sample absorbance readings at 517 nm for DPPH and at 734 nm for $ABTS^{•+}$. The spectra were measured using a quartz cell with an optical path of 1 cm.

The linearity of the Beer law was tested by performing 7-point calibration curves for DPPH and $ABTS^{•+}$ with ascorbic acid, using the respective methods employed in the assay runs carried out with the porphyrans. For a concentration range between 0 and 6 µg/mL of ascorbic acid, the linear regression equation obtained for the DPPH/ascorbic acid was y = -134.263 + 0.921314, with a R^2 of 0.999769. For $ABTS^{•+}$/ascorbic acid, the regression equation was y = -24282.0 + 0.836086, with a R^2 of 0.990831.

6. Suppression of the Na[FeEDTA]-catalysed oxidation of methyl red dye with H_2O_2

A stock solution of saturated methyl red dye in water was prepared by mixing 12.0 mg of the dye and 200 µL of acetic acid in 500.0 mL of distilled water. The suspension was vigorously stirred at room temperature for 1 h, after which the liquid was filtered to remove undissolved dye. Na[FeEDTA] (251.0 mg) was added to 250.0 mL of the dye stock solution and the mixture stirred till the complex dissolved completely.

The assay runs were carried out by charging a 1 cm optical path quartz cell with 1 mL of the of stock solution of the porphyran sample at 0.6 mg/mL, 1.8 mL of the Na[FeEDTA]/AcOH/ methyl red stock solution and 0.2 mL of 30% aqueous H_2O_2. The final concentration of porphyran in the cell was 0.2 mg/mL. The mixture was quickly but carefully stirred and electronic absorption spectra of the resulting solution were taken at 1 min intervals.

7. Suppression of the aerobic oxidation of methyl red dye with Na[FeEDTA]/AcOH/Benzaldehyde oxidant system

A 15 mL conical tube was charged with 2.9 mL of acetonitrile, 1.0 mL of stock solution of the porphyran sample at 0.8 mg/mL, 1.0 mL of the Na[FeEDTA]/AcOH/methyl red stock solution and 0.1 mL of benzaldehyde. The final concentration of porphyran in the test tube was 0.16 mg/mL. The tube was stoppered, and the mixture was stirred and left standing at room temperature. The electronic absorption spectrum was measured immediately after the preparation of the solution and after 400 min at room temperature.

8. Preparation of POR and POR-L2 films

The porphyran films were prepared according to reported methods, with adaptations [4].

A 50 mL conical plastic tube was charged with 20 mL of distilled water and 400 mg of porphyran. The polysaccharide was dissolved at 80 ºC with stirring. After dissolution, the resulting liquid was quickly filtered under vacuum with a quantitative filter paper, transferred to a conical plastic tube and quickly sonicated to remove trapped air bubbles. The solution was left standing for ca. 10 min at 80 ºC to further remove air bubbles. A 90 mm plastic Petri dish was pre-heated at 50 ºC on a hot plate and the solution of porphyran was then carefully poured into the Petri dish to cover the entire surface of the dish. The porphyran solution was heated at 50 ºC till dryness and the resulting film was carefully peeled from the dish surface with a pair of tweezers.

The preparation of the film with glycerol as plasticiser followed the same method using porphyran solution with 80 mg of glycerol (20% glycerol relative to the mass of polysaccharide used). For each composition film samples with around 1 cm^2 were randomly collected for analysis by scanning electron microscopy (FEG-SEM, JEOL

JSM7001F), coupled to energy dispersive spectroscopy (EDS, Oxford Instruments, Inca PentaFETx3). EDS elemental analysis was carried out upon uncoated material, while SEM observation and image collection took place after sample coating with Au-Pd alloy, to assure adequate electrical conductivity during observation. At least 3 random positions were studied in each sample for reproducibility.

9. Antimicrobiological activity assays

To evaluate the antimicrobial activity of the synthesized films, a modified microdilution technique (CLSI M7, 2006; and M38, 2008) was used. Overnight grown cultures of Escherichia coli (DSM-1103) and Staphylococcus aureus (DSM-1104), cultivated in Nutrient Agar (St. Louis, MO, USA), were dissolved in 0.85% NaCl solution and their concentration was adjusted to 1×10^{7} CFU.mL^{-1}. Exposure to the modified porphyran solutions was carried out in 200 uL, 96 well round-bottom microplates (Thermo Scientific), with a final inoculum concentration of 5×10^{5} CFU.mL^{-1} in Mueller-Hinton broth 2. Concentrations of the film solutions ranged from 1.6 to 0.2 mg/mL. Incubation lasted for 20 h at 35 °C, after which a reading of optical density at 625 nm was performed on a BioTek Instruments EPOCH 2 Microplate Reader. Three independent assays were performed, and the results presented in percentage of bacterial growth inhibition.

10. FTIR and NMR of POR-L2

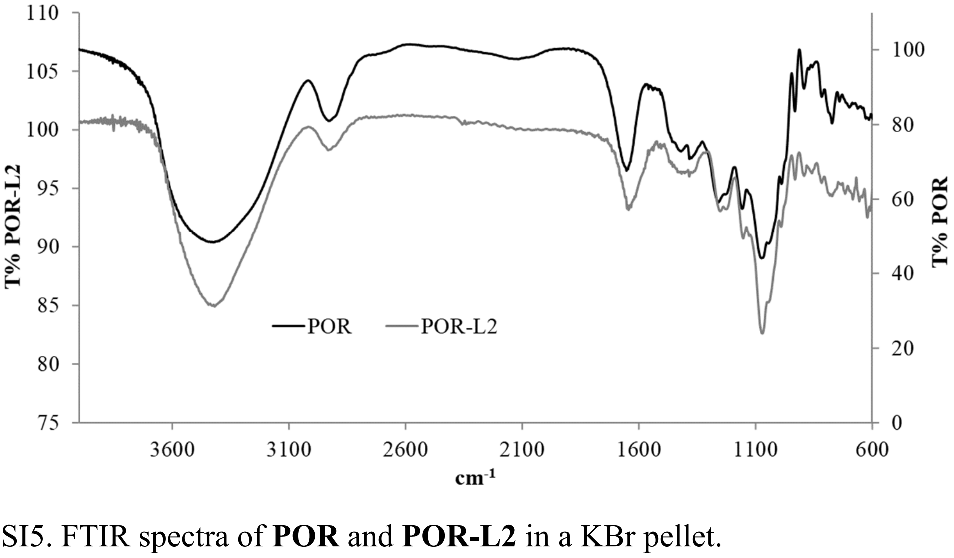

Figure SI5. FTIR spectra of **POR** and **POR-L2** in a KBr pellet.

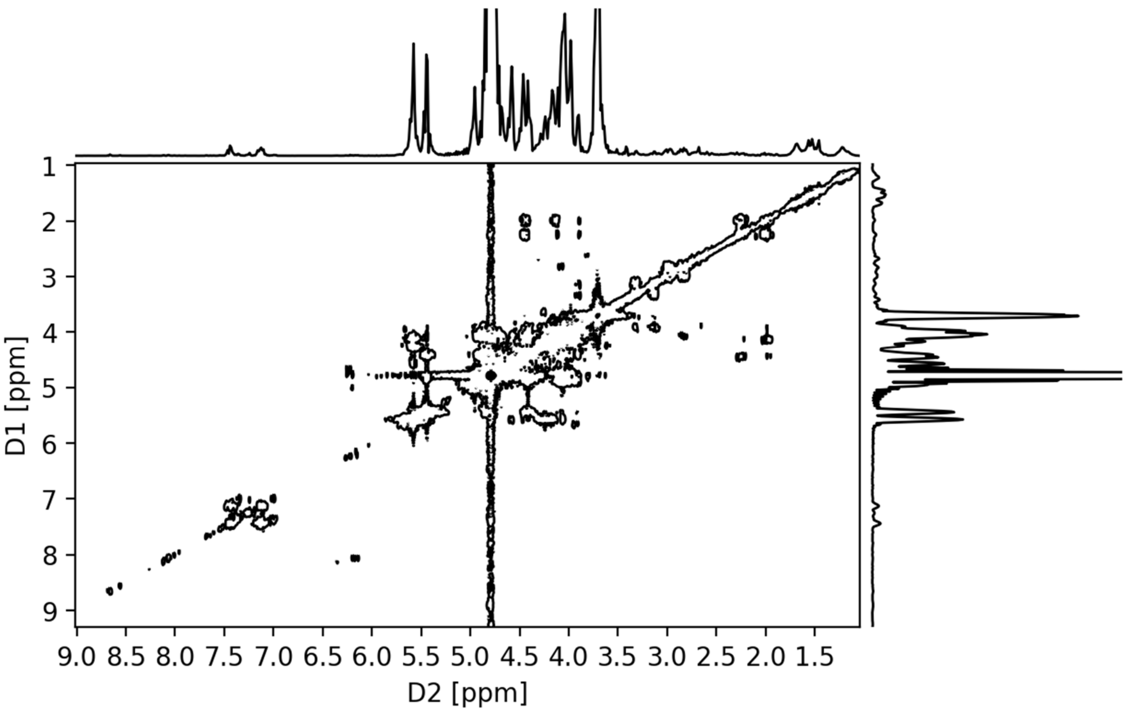

Figure SI6. ^{1}H-^{1}H TOCSY spectrum of **POR-L2**.

11. UV-Vis spectra of the ferric reducing power assays

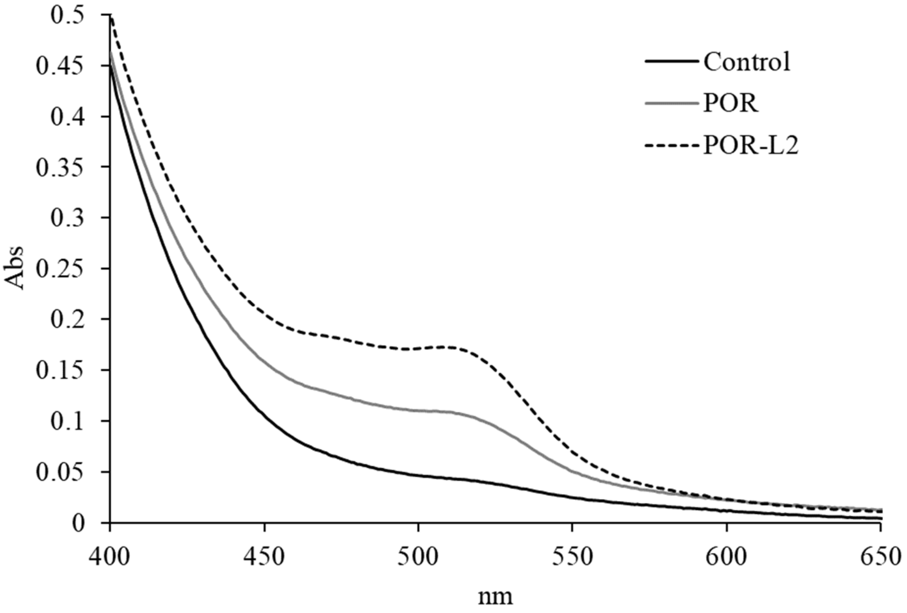

Figure SI7 – Electronic absorption spectra of the ferric reducing activity assays after 30 min. The control sample was $[Fe^{III}(Phen)_3]Cl_3$ in water at 0.55 mM (1.65×10^{-6} mol, 3 mL), in the absence of porphyrans. The concentration of the porphyran samples (**POR** and **POR-L2**) was 0.2 mg/mL.

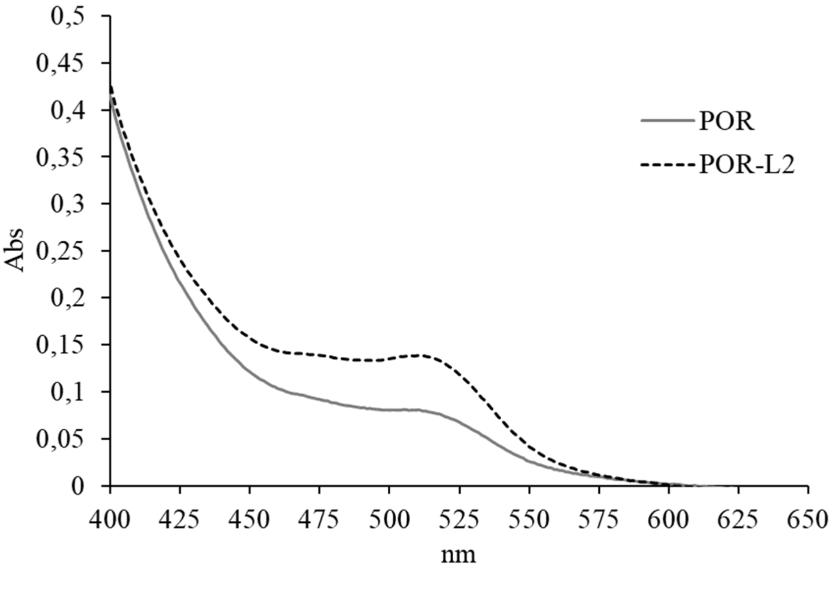

Figure SI8. Electronic absorption spectra of ferric reducing activity assays carried out with **POR** and **POR-L2,** after subtraction of the respective spectra at 0.2 mg, without 0.55 mM $[Fe^{III}(Phen)_3]Cl_3$.

Table SI 1. Obtained absorbances for the tested porphyrans after 30 min in the ferric reduction activity screening.

Sample [a]	t (min)	Abs 510 nm[b]
Control[c]	0	0.042 ± 0.003
	30	0.045 ± 0.002
**POR**[d]	30	0.108 ± 0.002
**POR**[e]	30	0.079 ± 0.002
**POR-L2**[d]	30	0.174 ± 0.002
**POR-L2**[e]	30	0.140 ± 0.002

[a] Concentration of **POR**s in the samples was 0.2 mg/mL and $[Fe^{III}(Phen)_3]Cl_3$ was 0.55 mM. Experiments were performed in triplicate using a quartz cell with a 1 cm optical path. [b] The obtained values are presented as mean ± SD. [c] The control sample was $[Fe^{III}(Phen)_3]Cl_3$ in water at 0.55 mM (1.65×10^{-6} mol, 3 mL).[d] Obtained absorbances without subtraction of the measured absorbances at 510 nm for the porphyrans at 0.2 mg/mL, in water. [e] The presented absorbances were obtained after subtraction with the measured absorbances at 510 nm for **POR** and **POR-L2** at 0.2 mg/mL, in water, as described in Section 3.3.

12. UV-Vis spectra of the DPPH and ABTS$^{\bullet+}$ assays

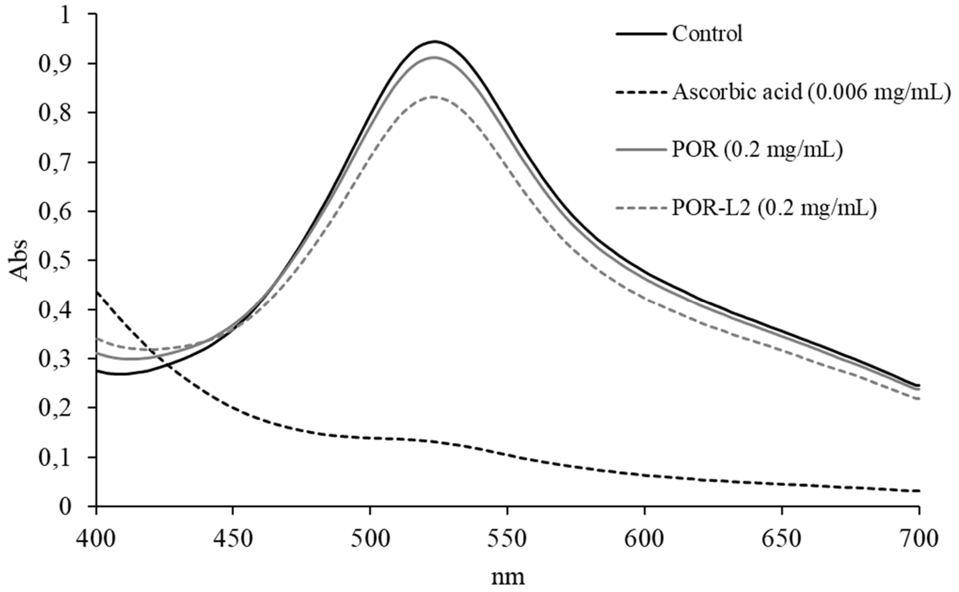

Figure SI9. Electronic absorption spectra of the DPPH$^{\bullet}$ radical in water and in the presence of either ascorbic acid, **POR** or **POR-L2**, after 30 min. The mass concentration of the PORs was 0.2 mg/mL. The spectra were measured with 1 cm optical path quartz cells.

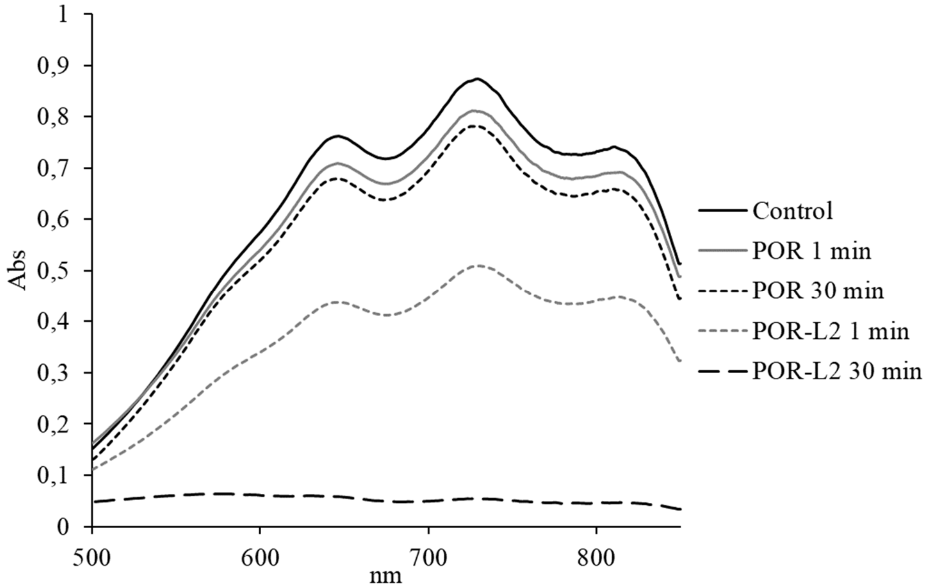

Figure SI10. Electronic absorption spectra of the ABTS$^{\bullet+}$ radical cation in water and in the presence of either **POR** or **POR-L2**. The mass concentration of the PORs was 0.2 mg/mL. The spectra were measured with a 1 cm optical path quartz cell.

13. UV-Vis spectra of the Na[FeEDTA]/AcOH/H_2O_2 and Na[FeEDTA]/AcOH/Benzaldehyde assays

A

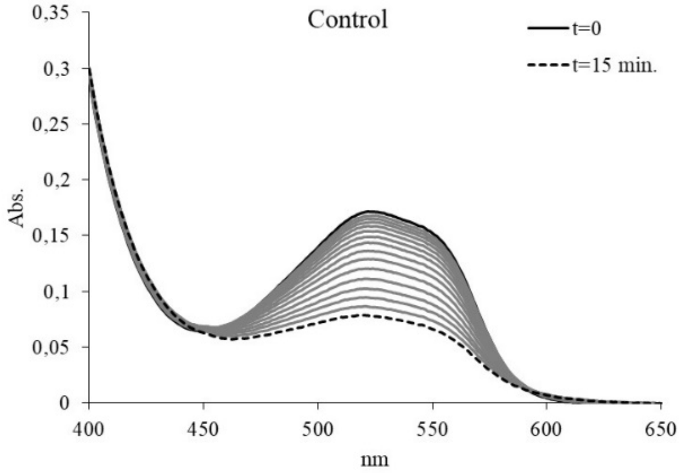

B

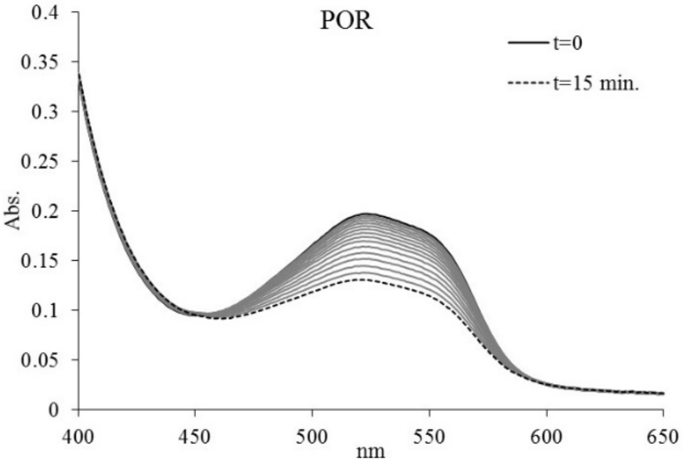

C

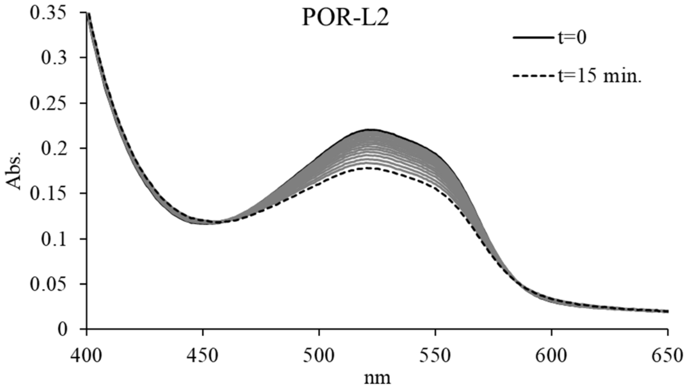

Figure SI11 – Electronic absorption spectra obtained for the *Na[FeEDTA]/AcOH/H_2O_2* assays: control run (A), **POR** (B) and **POR-L2** (C) after 15 min under the described oxidation conditions, at a mass concentration of 0.2 mg/mL in water. The spectra were measured with 1 cm optical path quartz cells.

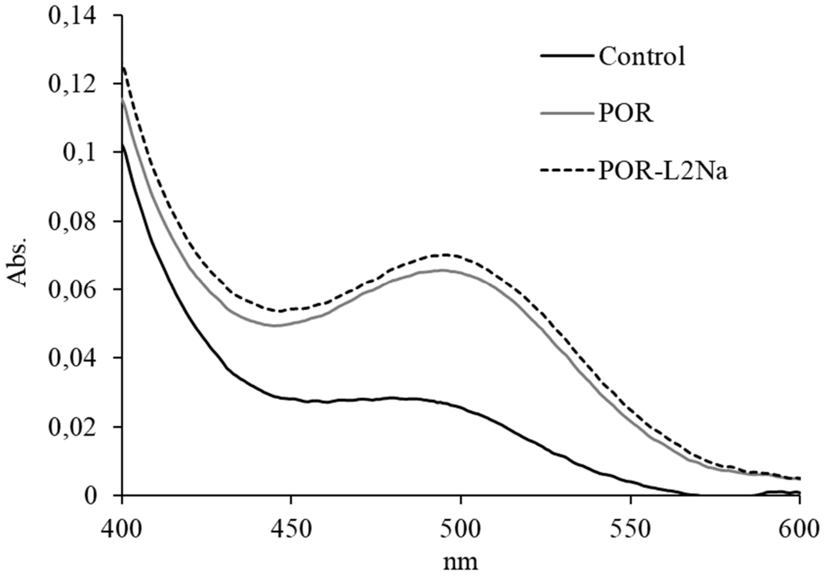

Figure SI12 – Electronic absorption spectra obtained for the *Na[FeEDTA]/AcOH/Benzaldehyde assays*, after 400 min under the described aerobic oxidation conditions, at a mass concentration of 0.16 mg/mL in acetonitrile/water (3:2). The control run was carried out with distilled water instead of the porphyran sample. The spectra were measured with a 1 cm optical path quartz cell.

14. Macrographs of the porphyran films

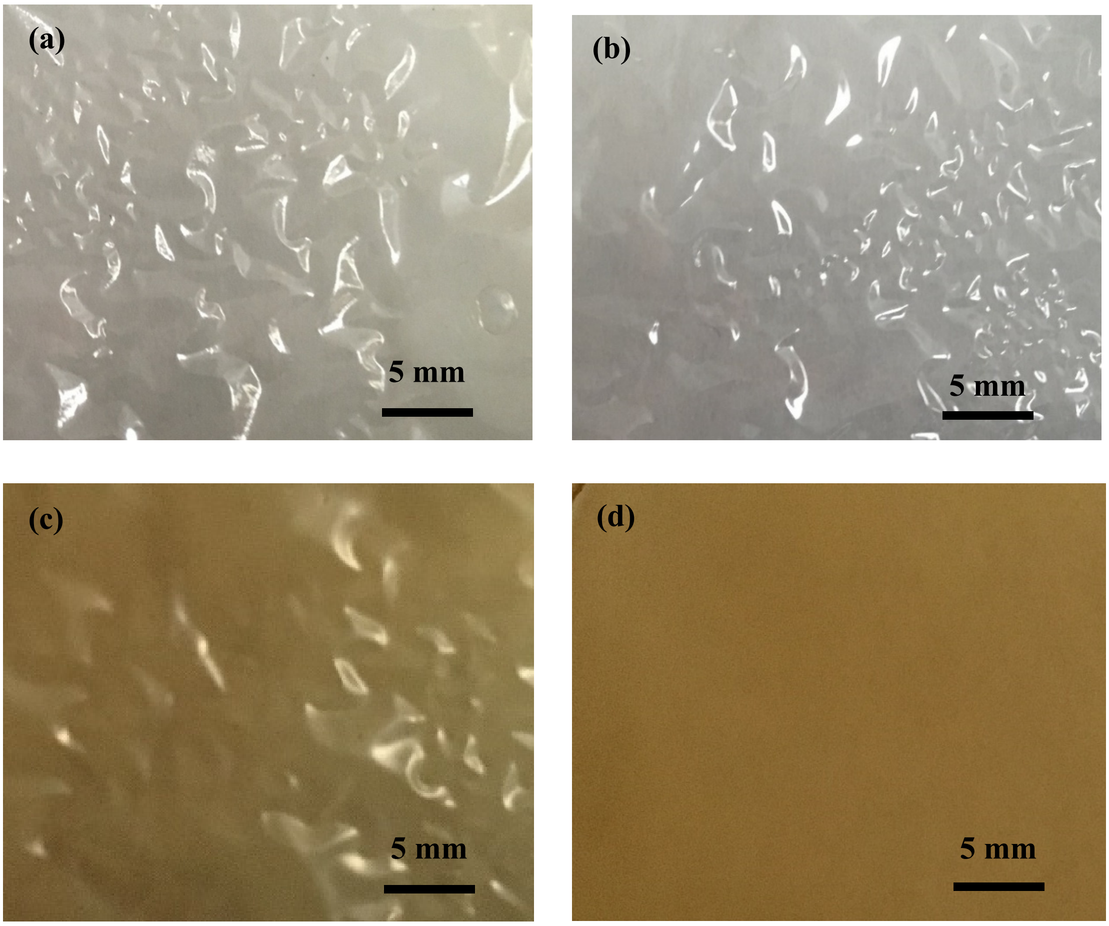

Figure SI13. Macrographs of the produced films: (a) **POR; (b)** POR/gly; (c) .**POR-L2**.; (d) **POR-L2**/gly

---

[1] M. Miceli, E. Roma, P. Rosa, M. Feroci, M.A. Loreto, D. Tofani, T. Gasperi, Synthesis of Benzofuran-2-One Derivatives and Evaluation of Their Antioxidant Capacity by Comparing DPPH Assay and Cyclic Voltammetry, Molecules. 23 (2018) 710. https://doi.org/10.3390/molecules23040710.

[2] R. Re, N. Pellegrini, A. Proteggente, A. Pannala, M. Yang, C. Rice-Evans, Antioxidant activity applying an improved ABTS radical cation decolorization assay, Free Radical Biology and Medicine. 26 (1999) 1231–1237. https://doi.org/10.1016/S0891-5849(98)00315-3.

[3] Z. Wang, Y. Lin, T. Li, F. Dai, G. Luo, G. Xiao, C. Tang, Phenolic profiles and antioxidant capacities of mulberry (Morus atropurpurea Roxb.) juices from different cultivars, International Journal of Food Properties. 22 (2019) 1340–1352. https://doi.org/10.1080/10942912.2019.1646272.

[4] R.E. Cian, P.R. Salgado, S.R. Drago, A.N. Mauri, Effect of glycerol and Ca+2 addition on physicochemical properties of edible carrageenan/porphyran-based films obtained from the red alga, Pyropia columbina, Journal of Applied Phycology. 27 (2015) 1699–1708. https://doi.org/10.1007/s10811-014-0449-5.
